# A New Inverse Probability of Selection Weighted Cox Model to Deal With Outcome‐Dependent Sampling in Survival Analysis

**DOI:** 10.1002/bimj.70056

**Published:** 2025-06-11

**Authors:** Vera H. Arntzen, Marta Fiocco, Inge M. M. Lakeman, Maartje Nielsen, Mar Rodríguez‐Girondo

**Affiliations:** ^1^ Mathematical Institute Section of Statistics Leiden University Leiden The Netherlands; ^2^ Department of Biomedical Data Sciences Section of Medical Statistics Leiden University Medical Center Leiden The Netherlands; ^3^ Department of Clinical Genetics Leiden University Medical Center Leiden The Netherlands; ^4^ Department of Human Genetics Leiden University Medical Center Leiden The Netherlands

**Keywords:** Cox regression, genetic epidemiology, outcome‐dependent sampling, survival analysis, weighting

## Abstract

Motivated by the study of genetic effect modifiers of cancer, we examined weighting approaches to correct for ascertainment bias in survival analysis. Outcome‐dependent sampling is common in genetic epidemiology leading to study samples with too many events in comparison to the population and an overrepresentation of young, affected subjects. A usual approach to correct for ascertainment bias in this setting is to use an inverse probability‐weighted Cox model, using weights based on external available population‐based age‐specific incidence rates of the type of cancer under investigation. However, the current approach is not general enough leading to invalid weights in relevant practical settings if oversampling of cases is not observed in all age groups. Based on the same principle of weighting observations by their inverse probability of selection, we propose a new, more general approach, called the generalized weighted approach. We show the advantage of the new generalized weighted cohort method using simulations and two real data sets. In both applications, the goal is to assess the association between common susceptibility loci identified in genome‐wide association studies (GWAS) and cancer (colorectal and breast) using data collected through genetic testing in clinical genetics centers.

**Abbreviations**
GWAS: genome‐wide association studies
BRCA: breast (and ovarian) cancer associated (genes)
reBias: relative Bias
MSE: mean‐squared error
CRC: colorectal cancer
SNP: single nucleotide polymorphism (genetic variant)

## 1 Introduction

Outcome‐dependent sampling is common in genetic epidemiology. Since harmful variants in cancer‐associated high‐risk genes are typically rare, an efficient sampling strategy to find carriers of these variants is to oversample affected individuals with a family history of a specific disease. For example, carriers of pathogenic variants in the Lynch syndrome‐associated gene *PMS2* and the breast‐ and ovarian cancer‐associated genes *BRCA1* and *BRCA2*, are often detected through genetic screening programs in which testing is targeted to families with multiple cases. Due to this testing strategy, the available study cohorts to investigate modifiers of cancer risk are often nonrepresentative samples of the target population (carriers of certain gene rare mutation). Carriers with an early diagnosis of cancer are more frequently included in the sampled population compared to those with delayed cancer diagnoses or individuals who remain disease‐free.

In the context of survival analysis, family‐based outcome‐dependent sampling results in an overrepresentation of events and short lifetimes, which without adjustment, leads to biased estimates of covariate effects when using, for example, a Cox proportional hazards model. This happens because the sampling mechanism affects the joint distribution of the time‐to‐event and covariate.

To solve this problem, two main approaches have been proposed in the literature: methods based on retrospective likelihood (Barnes et al. 2013; Carayol and Bonaïti‐Pellié 2004; Chatterjee et al. 2006) and the weighted cohort method (Antoniou et al. 2005) based on weighted Cox regression. The general idea of the methods based on retrospective likelihood is to formulate the likelihood of the observed covariate values conditional on the observed outcomes. These methods typically require to know the familial relations within the sample and the distribution of the covariate of interest, leading to analytically complex and computationally intensive methods. Without this information, when the overall age‐specific incidence rates in the population of interest are known, a widely used alternative approach to estimate the association between a set of covariates and time to cancer diagnosis under outcome‐dependent sampling is to use a weighted Cox regression model (Antoniou et al. 2005). The general idea is to propose a weighting scheme with different weights for affected (observed events) and unaffected (right‐censored) individuals according to an external source so that the resulting weighted sample mimics the true target population (Antoniou et al. 2005; Barnes et al. 2012) in terms of the age‐specific proportions of affected and unaffected individuals.

Due to its simplicity, this is an attractive approach. Related approaches have also been used in other fields. Weighting to handle informative sampling has also been investigated in the context of Cox proportional hazards models for double‐truncated data (Mandel et al. 2018; Rennert 2022), length‐biased sampling (Wang 1996), and in the context of augmented case–cohort settings (Barlow 1994; Therneau 1999). The integration of external survival information and sample data has been investigated to increase the efficiency of the Cox model in the context of randomly selected samples (Huang et al. 2016).

Despite its attractiveness, the weighted cohort method (Antoniou et al. 2005) has some limitations: it often leads to invalid weights in relevant practical situations since it is only workable under particular sampling schemes, such as those involving substantial oversampling of cases.

The primary objective of this study is to introduce a novel and more versatile inverse probability of selection weighting scheme, utilizing population‐based age‐specific incidence rates of the event of interest. This leads to the development of a generalized weighted cohort method capable of accommodating arbitrary levels of outcome‐dependent sampling, offering an improved alternative to the existing approach. As a secondary goal, we aim to conduct a sensitivity analysis to assess the performance of the weighted approaches in the presence of unobserved heterogeneity, particularly exploring within‐family correlations arising from shared, unobserved factors. Despite the frequent inclusion of multiple family members in studies employing the original weighted cohort method (see Table S1 for details), the influence of unobserved heterogeneity in this context remains unexplored. This aspect merits thorough investigation.

The rest of the paper is organized as follows. In Section 2, the commonly used weighted cohort Cox approach is revisited and its assumptions are discussed. A new alternative weighting scheme is proposed in Section 2.2. In Section 3, both weighting schemes are compared by means of an intensive simulation study. In Section 4, we present two real data illustrations. In both illustrations, the role of genetic variants as modifiers of cancer risk is studied using data sets of affected individuals and family members ascertained through genetic counseling in a clinical genetic center. In the first application, we focus on colorectal cancer (CRC) in carriers of the pathogenic variant *PMS2*, and in the second application, we analyze the association between a polygenic risk score (PRS) based on common breast cancer‐associated variants and breast cancer risk in multiple case families. Main conclusions, recommendations, and a final discussion follow in Section 5.

## 2 Weighted Cox Regression to Deal With Outcome‐Dependent Sampling

Let T be the time to event of interest, in age scale and assumed to be fully observed from birth (no left‐truncation), in the target population of interest. The typical target population in our context comprises individuals who are carriers of a specific rare mutation of interest. Denote by C the right‐censoring time, assumed to be uninformative. Denote by Z the covariate of interest. Since sampling schemes in genetic epidemiology are typically family‐based, denote the observed sample information by $\left(t_{ij},\delta_{ij},z_{ij}\right)$, where $i=1,\text{…},n_{j}$ index all included individuals in the sample belonging to family j. It is important to note that even though the sampling is family‐based, this does not necessarily imply the inclusion of multiple family members in the resulting sample. Assume that N families are observed, with varying observed size $n_{1},\text{…},n_{N}$, so that $n=\sum_{j=1}^{N}n_{j}$ individuals are included in the sample. The observed time to event for individual i in family j (denoted from now on by ij), is given by $t_{ij}=\min\left(T_{ij},C_{ij}\right)$. Define the noncensoring indicator $\delta_{ij}=I\left(T_{ij}\leq C_{ij}\right)$ where $\delta_{ij}$ is 1 if the event is observed or 0 if observation ij is right‐censored. $z_{ij}$ denotes covariate value for individual ij.

The observed data are collected through a family‐based outcome‐dependent sampling scheme. The selection process begins by testing the first individual in a family (the proband), focusing on those diagnosed with cancer at a young age and with a family history of cancer. If this initial individual tests positive for the mutation, the rest of the family is invited to participate in genetic testing. This approach may identify additional carriers of the mutation within the family, though this is not always the case. Consequently, due to variations in age and family history criteria across studies, influenced by disease severity and prevalence, the level of outcome‐dependent sampling varies across studies. Despite the diversity in the final configurations, samples of carriers of rare genetic variants obtained via genetic testing typically result in an overrepresentation of young cases in the sample. There is a connection between the sampling mechanism of genetic testing programs and right‐truncation. Probands are typically right‐truncated and the right‐truncation time can usually be defined. For example, if the criterion for getting genetically tested was being affected before the age of 60 years old, 60 would be the right‐truncation time. After identifying the proband, the rest of the family is invited to participate in the study. So, in principle the rest of participants are a randomly selected from the target population. However, there can be a further overrepresentation of young cases beyond the probands, making it difficult to formulate the right‐truncation variable for those individuals. Samples also typically include unaffected individuals, who are not right‐truncated and can be considered a random sample from the target population.

A common approach to estimate the effect of covariate Z on T is to use the Cox proportional hazards model with hazard function $h\left(t|z\right)=h_{0}\left(t\right)\exp\left(\beta z\right)$ where $h_{0}\left(t\right)$ is the baseline hazard. With prospective cohort data, the parameter β can be estimated maximizing the partial likelihood. However, the overrepresentation of events and short event times in the sample due to outcome‐dependent sampling affects the risk set composition along the follow‐up time in comparison to the true population, which may result in biased estimation of the covariate effect. In addition, the complex selection process does not allow for repairing biased sampling based solely on the sample information.

A possible solution to this problem is to consider a weighted Cox model using external information about the distribution of T in the population to construct weights reflecting individuals' selection probabilities.

### 2.1 The Weighted Cohort Approach Revisited

When T represents the age at cancer diagnosis, or another common disease, registry data about the marginal distribution of T in the target population are often accessible. In practical scenarios, the available external information is typically aggregated into K distinct age intervals, defined as $I_{1}=\left[a_{0},a_{1}\right)$, $I_{2}=\left[a_{1},a_{2}\right)$,…, $I_{K}=\left[a_{K-1},a_{K}\right)$. For cancer studies, the commonly available external data comprise the population cancer incidence rate $\mu_{k}$ for each age interval $I_{k}$. The seminal work of Antoniou et al. (2005) introduced a weighted Cox regression model with sampling weights derived in such a way that the incidence rates in each interval $I_{k}$, in the resulting pseudo‐population after weighting, align with the incidence rates $\mu_{k}$ in the target population.

However, before presenting the specific calculation of these weights, it is essential to acknowledge two main assumptions regarding the observed data in this context, given that the externally available data are discrete in time. First, constant hazards within each interval $I_{k}$ for k=1,…,K is assumed. Second, right‐censoring is also assumed to be discrete, occurring at the specified time points defining the intervals. This implies that if censored observations happen to fall within interval $I_{k}$, we assume that the censoring took place at point $a_{k}$. When these two prerequisites are met, the marginal distribution of T in the resulting weighted pseudo‐population, based on interval‐specific incidence rates, will follow the same distribution as in the reference population.

Let $r_{k}$ denote the number of individuals experiencing the event within the age interval, $I_{k}=\left[a_{k-1},a_{k}\right)$, k=1,…K. Similarly, $s_{k}$ denotes the number of individuals right‐censored within the age interval $I_{k}$ (i.e., follow‐up ends between age $a_{k-1}$ and $a_{k}$ without the event being observed). The term $p_{k}=\sum_{\left\{ij:t_{ij}\in I_{k},\delta_{ij}=1\right\}}t_{ij}$ denotes the total follow‐up time accumulated by all $r_{k}$ individuals experiencing the event in age interval $I_{k}$; the equivalent total follow‐up time accumulated by the $s_{k}$ right‐censored individuals is denoted by $q_{k}=\sum_{\left\{ij:t_{ij}\in I_{k},\delta_{ij}=0\right\}}t_{ij}$. Then, all $r_{k}$ cases in interval $I_{k}$ are assigned weight $w_{k}$ and all $s_{k}$ right‐censored individuals in interval $I_{k}$ are assigned weight $v_{k}$ such that

(1) $$\mu_{k}=\frac{w_{k}r_{k}}{w_{k}p_{k}+v_{k}q_{k}+\left(a_{k}-a_{k-1}\right)\sum_{l>k}\left(v_{l}s_{l}+w_{l}r_{l}\right)}.$$

In the right part of expression (1), the weighted total of affected observations is divided by the weighted total of observations at risk. Then, this weighted ratio is imposed to be equal to the population incidence rate $\mu_{k}$. As a result, after weighting the sample age‐specific incidence rates resemble the age‐specific incidence rates of the population.

However, since Equation (1) does not fully determine $w_{k}$ and $v_{k}$, the following constraint is incorporated to guarantee unique weights:

(2) $$\frac{w_{k}r_{k}+v_{k}s_{k}}{r_{k}+s_{k}}=1.$$

Combining Equations (1) and (2) provides unique expressions for $v_{k}$ and $w_{k}$:

(3) $$w_{k}=\frac{\mu_{k}\left(q_{k}\left(r_{k}+s_{k}\right)+\left(a_{k}-a_{k-1}\right)s_{k}\sum_{l>k}\left(r_{l}+s_{l}\right)\right)}{r_{k}s_{k}+\mu_{k}\left(q_{k}r_{k}-p_{k}s_{k}\right)},$$

where $\sum_{l>k}\left(r_{l}+s_{l}\right)$ are all observations in age groups older than k. The weight equation for censored individuals is given by

(4) $$v_{k}=\frac{1}{s_{k}}\left(r_{k}+s_{k}-w_{k}r_{k}\right).$$

Once weights $v_{k}$, $w_{k}$ for each age interval $I_{k}$, k=1,…,K are calculated, the regression parameter β can be estimated using the following weighted score equation:

(5) $$U_{a}\left(\beta \right)=\sum_{ij:\delta_{ij}=1}z_{ij}-\sum_{ij:\delta_{ij}=1}\frac{\sum_{l\varepsilon R(t_{ij})}W_{l}z_{l}\exp\left[\beta z_{l}\right]}{\sum_{l\varepsilon R(t_{ij})}W_{l}\exp\left[\beta z_{l}\right]},$$

where $R(t_{ij})$ is the set of individuals still at risk just before $t_{ij}$, that is, $R\left(t_{ij}\right)=\left\{l:t_{ij}\leq t_{l}\right\}$, and weight $W_{ij}$ for individual ij ($i=1,\text{…},n_{j}$, j=1,…,N) is defined as

(6) $$W_{ij}=\left\{\begin{array}{cc} w_{k}, & \;\text{if}\;\delta_{ij}=1\;\;\text{and}\;\;t_{ij}\in \left[a_{k-1},a_{k}\right)\\ v_{k}, & \;\text{if}\;\delta_{ij}=0\;\;\text{and}\;\;t_{ij}\in \left[a_{k-1},a_{k}\right). \end{array}\right.$$

The derivation and justification of unbiasedness of the estimator resulting from expression (5) are outlined in Appendix A1. We followed the same reasoning presented by Mandel et al. (2018), who introduced an inverse probability weighted Cox model to address double truncation. According to the authors, their findings extend beyond the context of double truncation to deal with a broader range of biased sampling scenarios. Crucially, in our setting, we assume conditional independence between the selection event and covariate values, given the observed event time. This assumption allows us to directly apply the results of Mandel et al. (2018), with the selection event treated as a function of the observed event times. Moreover, since weights $\hat{W}$ are estimated, several regularity conditions need to be made to guarantee the large sample properties of our method. Following Mandel et al. (2018) again, and under the same regularity conditions and two conjectures for $\hat{W}$, the solution of (5) with $\hat{W}$ plugged in is consistent and asymptotically normal. These two required conjectures refer to the uniform convergence and iid representation of $\hat{W}$ and have been proven to hold in the double truncation setting (de Uña Álvarez and Van Keilegom 2021). In principle, the asymptotic analysis developed by de Uña Álvarez and Van Keilegom (2021) applies to any general biased sampling setting, including ours. However, it is important to note that the particular form of the sampling probabilities is critical to ensure the existence of a nonparametric maximum likelihood estimator. In very special selection schemes, regularity conditions 1–4, especially condition 3, regarding the positivity assumption about the weight function, might be violated, and hence the results from Mandel et al. (2018) and de Uña Álvarez and Van Keilegom (2021) would not apply. To elaborate further, Appendix A1 provides a detailed explanation.

A number of conditions are required to guarantee finite and positive weights $w_{k}$ and $v_{k}$, namely:

(7) $$r_{k}>0$$

(8) $$s_{k}>0$$

(9) $$r_{k}>\mu_{k}p_{k}\frac{s_{k}}{s_{k}+\mu_{k}q_{k}}$$

(10) $$w_{k}<1+\frac{s_{k}}{r_{k}}.$$

Conditions (7) and (9) are required to get proper $w_{k}$ weights for the cases, while conditions (8) and (10) are required to get valid $v_{k}$ weights for those that are censored. Condition (7) implies the observation of events in all the considered intervals while condition (8) implies the presence of right‐censored observations in all intervals under consideration. Conditions (9) and (10) are more difficult to interpret and evaluate beforehand, but they are both related to the level of oversampling of events. As discussed by Antoniou et al. (2005), if oversampling of events occurs in all considered age groups both conditions are typically fulfilled. However, as we will show in our real data application, oversampling of young cancer cases is the norm in genetic epidemiology, but not necessarily the case at older ages, so condition (9) and especially (10) might not be fulfilled in relevant practical scenarios.

Under oversampling of events at interval $I_{k}$, condition (9) is verified since $r_{k}>\mu_{k}p_{k}$, that is, the observed number of events in interval $I_{k}$ is larger than the expected number of events assuming the population incidence rate ($\mu_{k}$). Actually, since $\mu_{k}q_{k}$ is usually positive, $0<\frac{s_{k}}{s_{k}+\mu_{k}q_{k}}<1$ in general which implies that condition (9) is fulfilled even when no oversampling of events is observed in interval $I_{k}$. However, condition (10) is cumbersome. Since it involves the estimated weight for events $w_{k}$ together with the ratio of events and right‐censored observations in interval $I_{k}$ ($\frac{s_{k}}{r_{k}}$), this condition is often not satisfied when there is no clear oversampling of cases in interval $I_{k}$. In such situations, $w_{k}$ can still be calculated but it becomes small, which leads to violating condition (10).

When any of the conditions (7)–(10) are not satisfied, the weighted cohort method can still be applied by merging intervals, however the method becomes then less precise and dependent on sample‐specific characteristics which may hamper comparability among studies using this method.

We therefore propose an alternative, more general weighting scheme which allows to overcome the aforementioned limitations.

### 2.2 The New Generalized Weighted Cohort Approach

We propose a new weighting scheme to correct outcome‐dependent sampling using external information. In contrast to the previous weighted cohort method, the new approach is more general, as it can be applied with arbitrary levels of over or underrepresentation of events.

Similar to the original method, the newly proposed weights represent sampling probabilities given the observed time to event of each individual so that the resulting pseudo‐population matches the target population of reference in terms of the marginal distribution of T. It also relies on a piecewise constant hazard and discrete right‐censoring assumptions. The same score function (5) and justification of its validity apply. However, here we take a different approach to derive the weights. Instead of directly using the incidence rates, we focus on the risk sets at the beginning at each interval $I_{k}$ and weight the individuals so that the resulting weighted risk set presents the same ratio of events and nonevents as one would expect if the sample would have been randomly drawn from the target population.

Let $N_{k}$ denote the number of individuals at risk (those who have not experienced the event yet) at the beginning of the interval $I_{k}$ in our sample, denoted by $S_{O}$, potentially drawn under an outcome‐dependent sampling mechanism. Now denote by $S_{P}$ a hypothetical random sample of the target population with the same $N_{k}$ number of individuals at risk at the beginning of the interval $I_{k}$. In both cases, $N_{k}$ can be split into two disjoint parts: (a) the number of individuals that experience the event within the interval $I_{k}$ and (b) those experiencing the event in later intervals. However, if $S_{O}$ is obtained using outcome‐dependent sampling, the expected number of individuals belonging to each of these two parts in $S_{O}$ and $S_{P}$ will, in general, differ.

For the hypothetical random sample $S_{P}$, $N_{k}$ can be decomposed as follows:

(11) $$N_{k}=N_{k}S_{k}+N_{k}\left(1-S_{k}\right),$$

where $S_{k}=P\left(T\geq a_{k}|T\geq a_{k-1}\right)$ represents the conditional probability of experiencing the event in a later time interval than interval $I_{k}$ given that the event has not been experienced before interval $I_{k}$ in the reference population. $S_{k}$ can be directly calculated from the typically available population cancer incidence rates $\mu_{k}$ for each age interval $I_{k}$, since $S_{k}=e^{-\mu_{k}\left(a_{k}-a_{k-1}\right)}$, k=1,…K, assuming, as previously discussed, constant hazards within each interval $I_{k}$. Accordingly, $1-S_{k}$ is the probability of experiencing the event in the interval $I_{k}$ given that it has not been experienced before, in the reference population. From expression (11) follows that the ratio between events and nonevents in interval $I_{k}$ in the reference population is given by $\frac{1-S_{k}}{S_{k}}$. Under the assumption of constant hazards within each prespecified interval $I_{k}$, the ratio of events to nonevents completely determines the incidence rate in interval $I_{k}$, thereby entirely characterizing the marginal distribution of T.

The same decomposition of the risk set at the beginning of interval $I_{k}$ can be made for the observed sample $S_{O}$, potentially subject to outcome‐dependent sampling:

(12) $$N_{k}=N_{k}S_{k}^{o}+N_{k}\left(1-S_{k}^{o}\right),$$

where $S_{k}^{o}$ is the observed proportion of individuals at risk at time $a_{k-1}$ experiencing the event beyond $I_{k}$, calculated with the sample data.

In our new approach, we keep those subjects nonexperiencing the event at interval $I_{k}$ unweighted ($v_{k}=1$) while we assign specific weights ($w_{k}$) to those subjects experiencing the event of interest in interval $I_{k}$ making use of the decompositions given by expressions (11) and (12). Specifically, weights $w_{k}$ correct the oversampling (or undersampling) of cases, such that the ratio between events and nonevents on the interval $I_{k}$ in the resulting pseudo‐population after weighting is the same as in the reference population:

(13) $$w_{k}=\frac{\left(1-S_{k}\right)}{S_{k}}\frac{S_{k}^{o}}{\left(1-S_{k}^{o}\right)}.$$

Equation (13) illustrates that the population ratio between events and nonevents within interval $I_{k}$, denoted as $\frac{1-S_{k}}{S_{k}}$, is multiplied by the inverse quantity based on the observed data, $\frac{1-S_{k}^{o}}{S_{k}^{o}}$. After weighting, the composition of the risk set within interval $I_{k}$, in terms of the ratio of events to nonevents, resembles the composition of the risk set within interval $I_{k}$ in the reference population. As a result, under oversampling of cases, weights for affected individuals in interval $I_{k}$ are $w_{k}<1$, representing the inverse of the probability of being selected. Alternatively, under undersampling of cases, $w_{k}>1$. Interestingly, in the absence of outcome‐dependent sampling, that is, under random sampling, $w_{k}=1$ and the new generalized weighted cohort method coincides with the regular unweighted Cox model. Note that these results rely on the aforementioned assumptions of piecewise constant hazards and discrete right‐censoring.

With our new proposal, two conditions need to be fulfilled in order to get valid weights: $(1-S_{k}^{o})>0$, k=1,…,K and $S_{K}>0$. The first condition $(1-S_{k}^{o})>0$ is satisfied if events are observed in each interval $I_{k}$, so as the original weighted cohort method, observation of events in all group ages is a requirement of the new generalized weighted cohort method. However, the new generalized weighted cohort method does not require the presence of right‐censoring which makes it a more general and natural approach. The condition $S_{K}>0$ only involves the last interval and implies that the method is suitable for studying events not experienced by a part of the population during the relevant follow‐up time. This is a mild condition that is always satisfied when studying defective distributions (S(∞)>0) such as time to cancer or other diseases since not all population members will develop the event of interest. Even if our interest would be to study time to death or the target population would be a highly susceptible population to a specific cancer with lifetime risk of 1, the new weights could still be applied with an appropriate choice of the upper limit of the last interval K.

Once the weights are calculated, the regression parameter β can be estimated using the weighted score equation (5) and robust estimates of the standard errors can be obtained using a sandwich estimator, as proposed by Antoniou et al. (2005) in the original weighted cohort approach.

In summary, both the existing weighted cohort and the new generalized weighted cohort approaches generate pseudo‐populations by means of inverse probability of selection weighting, but these pseudo‐populations are different. The newly proposed generalized weighted cohort method is more general since it does not require oversampling or undersampling of events in all or at specific intervals and makes less assumptions about the level of right‐censoring.

## 3 Simulation Study

A simulation study was conducted with the primary aim of assessing the new generalized weighted cohort method's performance and comparing it with the existing approach in several scenarios intended to mimic relevant situations in practice. A secondary aim is to assess the sensitivity of the weighted methods to misspecification due to the presence and failure to adjust for unobserved familial heterogeneity.

We consider two data‐generating mechanisms, each to study each of the aforementioned primary and secondary aims.

### 3.1 Simulation Setup I

To address our primary aim, simulated data were generated using the following model:

(14) $$\lambda_{ij}\left(t\right)=u_{j}\lambda_{0}\exp\left(\beta z_{ij}\right),$$

where t is the observed event time, $\lambda_{0}=\frac{1}{60}$ represents the constant baseline hazard, Z is a continuous covariate assumed to be normally distributed (Z∼N(0,1)), and β represents the associated log‐hazard ratio. We assumed follow‐up stops at 100 years, administratively censoring all individuals with events occurring thereafter. Moreover, we considered the addition of random censoring during follow‐up, sampled from an exponential distribution $C∼\text{exp}(c_{0})$). We considered three scenarios regarding random censoring: no random censoring, medium level of random censoring ($c_{0}=60$), and high level of random censoring (($c_{0}=40$)). The family size in the population is set to $n_{j}$ (family size of size $n_{j}=2$ and 5 members were considered). In each scenario, for each of the M=1000 Monte Carlo trials, we generated N families (N = 250, 500, 750). Family‐based outcome‐dependent sampling was implemented by including families in the sample if for at least $n_{A}$ family members the event was observed before the end of follow‐up ($n_{A}=1,3$). The different combinations of $n_{A}$ and $n_{j}$ lead to three different scenarios with increasing level of outcome‐dependent sampling: scenario 1 (A1) with $n_{j}=5$ and $n_{A}=1$ represents the mildest level of selection, scenario 2 (A2) with $n_{j}=5$ and $n_{A}=3$ represents a medium level of outcome‐dependent selection, and scenario 3 (A3) with $n_{j}=2$ and $n_{A}=1$ represents the strongest level of outcome‐dependent sampling in the simulation study. Moreover, all included families had at least one “young affected” defined as having observed event time smaller than the first quartile of simulated T distribution. This mimics the common practice in clinical genetics centers: families are invited to participate in genetic studies when a young family member is diagnosed with the event at a young age. For each of the three considered number of generated families, N, the combination of the level of outcome‐dependent sampling and random censoring level resulted in a different number of included families. The mean number of included families across the M=1000 Monte Carlo trials is denoted by $\bar{N}$. In terms of covariate effect, the null case (β=0) and two alternative scenarios (β=0.3,1) were considered.

For each considered value of β, the underlying population was generated by simulating a large data set (N = 200,000, $n_{j}=1$) without ascertainment and it was used to approximate the population hazards needed to calculate the weights. In all simulation scenarios, we considered five age intervals, each with a width of 20 years.

Our estimand is the log‐hazard ratio β which represent the covariate effect. Each simulated data set is analyzed in three ways, using a naive unweighted Cox model, the traditional weighted cohort approach, and the new generalized weighted cohort. As performance measures, we assess relative bias across M=1000 Monte Carlo trials (defined as the difference between the simulated mean and true parameter value divided by the true value), mean square error, and coverage proportions of the 95% confidence intervals, constructed based on a normal approximation. Standard errors of β were computed as the square root of the diagonal elements of the observed Hessian matrix for the unweighted approach. For the weighted approaches, robust estimates of the standard errors were obtained using the Huber–White sandwich estimator (Huber 1967; White 1980). For the null case with β=0, bias is reported instead of relative bias, not defined in this case. Moreover, the proportion of negative weights in the M=1000 Monte Carlo trials is reported for the traditional weighted cohort method.

### 3.2 Simulation Setup II

To address our secondary aim of assessing the sensitivity of the weighted methods to misspecification due to the presence of familial heterogeneity, we consider a second simulation setting with a slightly different data generation mechanism. In the previous simulation setting, we have assumed, as the proposed models in Section 2, that differences among individuals in terms of hazards can be fully accounted for by including covariates in the Cox proportional hazards model. However, when samples contain multiple members of the same family (often the case when applying the traditional weighted cohort approach as shown in Table S1), unmeasured heterogeneity may arise since members of the same family often share common unmeasured characteristics such as genetic, social, dietary, or other factors. In this second simulation setting, to introduce such unmeasured heterogeneity in the simulated data, we consider an extension of the data generation model specified in expression (14) by adding a latent (frailty) term, U, shared by all members of the same family:

(15) $$\lambda_{ij}\left(t\right)=u_{j}\lambda_{0}\exp\left(\beta z_{ij}\right),$$

where $u_{j}∼\Gamma \left(1,\theta \right)$ is a latent term (frailty), shared by the $n_{j}$ members of a given family j. The larger the value of the variance θ, the more family members are alike and the larger the difference between families, yielding larger unobserved family effects. We consider two different values of within‐family correlation: “low” (θ = 0.1) and “large” (θ=1). Note that the latent frailty U and the covariate under investigation, Z are independent. Our estimand is the conditional log‐hazard ratio β. We expect that, as in the traditional unweighted Cox regression context (Henderson and Oman 1999), the presence of U but it being ignored introduces bias in the estimation of β using weighted Cox regression due to noncollapsability, even when U is independent of the covariate of interest Z. However, we also expect that in the presence of outcome‐dependent sampling and with the use of inverse probability of selection weighted Cox models the bias in the estimation of β is smaller than in the usual case based on random sampling.

The rest of the simulation specifications were as in the previous Simulation I, except N, the number of families was fixed to 500 in this setting and the level of random censoring fixed at medium level (C∼exp(60)). For each scenario, weighted Cox models were estimated using the traditional and the generalized weighting scheme. Results obtained with the standard choices of using an unweighted Cox model or a shared gamma frailty model (unweighted) are also reported. Note that directly applying the proposed weights to weight individuals and fitting a regular frailty model is not considered since the key assumption of independence between event times and selection given the covariate is violated in the presence of unobserved heterogeneity. In each interval, observed differences in event times are attributable to both the covariate under investigation and the unobserved frailty term.

### 3.3 Simulation Results

#### 3.3.1 Simulation I

**FIGURE 1:**
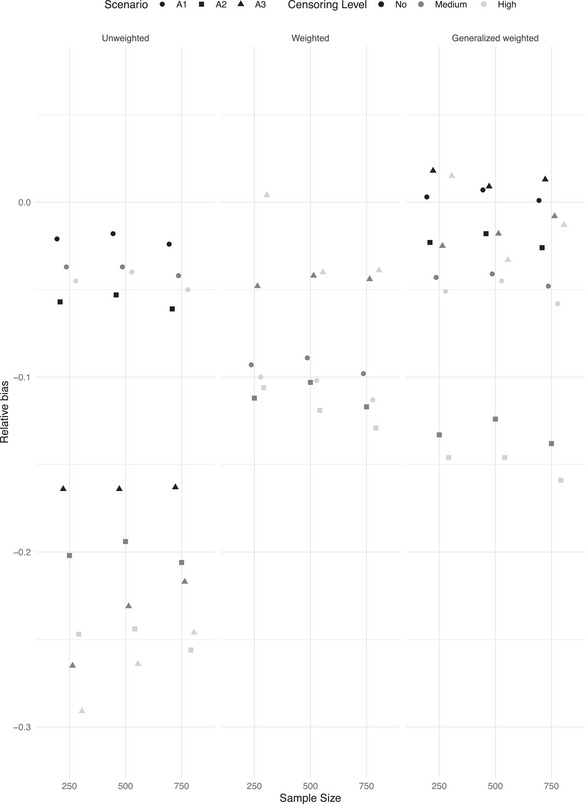
Simulation I. Relative bias (reBias) for $\hat{\beta }=0.3$ along 1000 trials for different levels of random right‐censoring, ascertainment, and sample sizes. A1: mild level of ascertainment; A2: medium level of ascertainment; A3: strong level of ascertainment.

We first present the results obtained when data are generated under the assumption of fully observed heterogeneity. Table 1 (no random censoring scenario), Table 2 (medium level of random censoring), and Table 3 (high level of random censoring) contain the results in terms of relative bias, MSE, and coverage probabilities for the three studied methods, unweighted Cox, weighted cohort, and the new generalized weighted cohort method. For the weighted cohort approach, we additionally provide the percentage of invalid weights across the M=1000 Monte Carlo trials. The new generalized weighted cohort method does not suffer from this problem, and it does not provide negative weights. In addition, Figure 1 (weak covariate effect) and 2 (strong covariate effect) summarize the behavior of the three studied methods in Simulation I across different levels of censoring and outcome‐dependent sampling levels in terms of relative bias. The level of outcome‐dependent sampling, the level of right‐censoring, and the covariate effect size determine the observed differences among the studied methods, driven by the differences in relative bias and the proportion of invalid weights in the weighted cohort method.

**TABLE 1:** Simulation I. Relative bias (reBias), mean square error (MSE), and coverage probability (Coverage) for $\hat{\beta }$ along 1000 trials. A1: mild level of ascertainment; A2: medium level of ascertainment; A3: strong level of ascertainment; N: number of families. The proportion of invalid (negative) weights along 1000 trials is also reported for the weighted cohort approach. The generalized weighted cohort approach did not lead to negative weights. No random censoring.

					Unweighted			Weighted cohort				Generalized weighted cohort		
β	Scenario	N	$\bar{N}$	% cens	reBias	MSE	Coverage	reBias	MSE	Coverage	Invalid weights	reBias	MSE	Coverage
β=0	A1	250	191	17	0.001	0.001	0.937	—	—	—	—	0.001	0.001	0.935
		500	381		0.001	0.001	0.938	—	—	—	—	0.001	0.001	0.941
		750	572		<0.001	<0.001	0.945	—	—	—	—	<0.001	<0.001	0.952
	A2	250	185	15	0.001	0.001	0.939					0.001	0.001	0.941
		500	369		0.001	0.001	0.942	—	—	—	—	0.001	0.001	0.946
		750	553		−0.001	<0.001	0.944	—	—	—	—	<0.001	<0.001	0.943
	A3	250	109	11	<0.001	0.005	0.943	—	—	—	—	−0.002	0.009	0.926
		500	219		0.001	0.002	0.962	—	—	—	—	<0.001	0.004	0.949
		750	328		0.001	0.002	0.954	—	—	—	—	0.001	0.003	0.943
β=0.3	A1	250	191	18	−0.021	0.001	0.925	—	—	—	—	0.003	0.001	0.939
		500	382		−0.018	0.001	0.939	—	—	—	—	0.007	0.001	0.955
		750	572		−0.024	0.001	0.933	—	—	—	—	0.001	<0.001	0.947
	A2	250	184	16	−0.057	0.002	0.904		—	—	—	−0.023	0.002	0.933
		500	367		−0.053	0.001	0.896	—	—	—	—	−0.018	0.001	0.937
		750	551		−0.061	0.001	0.859	—	—	—	—	−0.026	0.001	0.938
	A3	250	109	11	−0.164	0.007	0.892	—	—	—	—	0.018	0.009	0.941
		500	219		−0.164	0.005	0.847	—	—	—	—	0.009	0.004	0.940
		750	328		−0.163	0.004	0.777	—	—	—	—	0.013	0.003	0.949
β=1	A1	250	191	23	−0.034	0.003	0.858	—	—	—	—	0.008	0.002	0.947
		500	381		−0.035	0.002	0.780	—	—	—	—	0.007	0.001	0.960
		750	573		−0.037	0.002	0.687	—	—	—	—	0.005	0.001	0.951
	A2	250	176	20	−0.069	0.006	0.662	—	—	—	—	−0.018	0.003	0.930
		500	353		−0.070	0.006	0.392	—	—	—	—	−0.020	0.001	0.910
		750	529		−0.072	0.006	0.205	—	—	—	—	−0.021	0.001	0.881
	A3	250	109	15	−0.205	0.048	0.264	—	—	—	—	0.030	0.013	0.937
		500	219		−0.205	0.045	0.055	—	—	—	—	0.026	0.006	0.934
		750	328		−0.206	0.044	0.009	—	—	—	—	0.023	0.004	0.946

**TABLE 2:** Simulation I. Relative bias (reBias), mean square error (MSE), and coverage probability (Coverage) for $\hat{\beta }$ along 1000 trials. A1: mild level of ascertainment; A2: medium level of ascertainment; A3: strong level of ascertainment; N: number of families. The proportion of invalid (negative) weights along 1000 trials is also reported for the weighted cohort approach. The generalized weighted cohort approach did not lead to negative weights. Medium level of random censoring (C∼exp(60)).

					**Unweighted**			**Weighted cohort**				**Generalized weighted cohort**		
β	Scenario	N	$\bar{N}$	% cens	reBias	MSE	Coverage	reBias	MSE	Coverage	Invalid weights	reBias	MSE	Coverage
β=0	A1	250	122	44	<0.001	0.003	0.943	−0.001	0.003	0.948	0.000	<0.001	0.003	0.949
		500	243		<0.001	0.002	0.944	<0.001	0.001	0.948	0.000	<0.001	0.002	0.940
		750	365		−0.001	0.001	0.943	−0.001	0.001	0.941	0.000	−0.001	0.001	0.948
	A2	250	74	31	<0.001	0.004	0.934	−0.001	0.006	0.925	0.003	<0.001	0.005	0.933
		500	148		0.002	0.002	0.939	0.001	0.003	0.946	0.000	0.002	0.003	0.939
		750	222		−0.001	0.001	0.939	−0.001	0.002	0.931	0.000	−0.001	0.002	0.938
	A3	250	59	28	0.004	0.013	0.942	0.001	0.032	0.931	0.200	0.005	0.038	0.908
		500	117		0.001	0.006	0.955	<0.001	0.012	0.945	0.070	−0.001	0.014	0.931
		750	176		0.002	0.004	0.949	0.001	0.008	0.944	0.018	0.001	0.009	0.945
β=0.3	A1	250	124	44	−0.037	0.003	0.940	−0.093	0.004	0.910	0.000	−0.043	0.004	0.939
		500	247		−0.037	0.002	0.934	−0.089	0.002	0.887	0.000	−0.041	0.002	0.935
		750	370		−0.042	0.001	0.922	−0.098	0.002	0.846	0.000	−0.048	0.001	0.923
	A2	250	75	31	−0.202	0.008	0.813	−0.112	0.007	0.896	0.003	−0.133	0.007	0.885
		500	150		−0.194	0.005	0.733	−0.103	0.004	0.901	0.000	−0.124	0.004	0.871
		750	225		−0.206	0.005	0.584	−0.117	0.003	0.866	0.000	−0.138	0.003	0.821
	A3	250	60	27	−0.265	0.018	0.878	−0.048	0.030	0.920	0.185	−0.025	0.035	0.926
		500	119		−0.231	0.010	0.861	−0.042	0.012	0.941	0.056	−0.018	0.015	0.933
		750	179		−0.217	0.008	0.809	−0.044	0.008	0.948	0.020	−0.008	0.009	0.947
β=1	A1	250	138	44	−0.063	0.007	0.779	−0.039	0.006	0.912	0.000	−0.003	0.005	0.946
		500	276		−0.064	0.006	0.631	−0.039	0.003	0.873	0.000	−0.004	0.002	0.954
		750	414		−0.065	0.005	0.496	−0.041	0.003	0.807	0.000	−0.005	0.002	0.944
	A2	250	83	31	−0.173	0.034	0.259	−0.045	0.008	0.901	0.000	−0.061	0.010	0.869
		500	167		−0.173	0.032	0.042	−0.047	0.005	0.887	0.000	−0.063	0.007	0.793
		750	250		−0.175	0.032	0.005	−0.050	0.005	0.815	0.000	−0.065	0.006	0.705
	A3	250	69	28	−0.307	0.107	0.177	0.005	0.034	0.908	0.083	0.027	0.046	0.882
		500	138		−0.282	0.085	0.041	−0.004	0.015	0.926	0.016	0.027	0.020	0.927
		750	206		−0.272	0.078	0.011	−0.003	0.010	0.941	0.003	0.035	0.013	0.923

**TABLE 3:** Simulation I. Relative bias (reBias), mean square error (MSE), and coverage probability (Coverage) for $\hat{\beta }$ along 1000 trials. A1: mild level of ascertainment; A2: medium level of ascertainment; A3: strong level of ascertainment; N: number of families. The proportion of invalid (negative) weights along 1000 trials is also reported for the weighted approaches. High level of random censoring (C∼exp(40)).

					**Unweighted**			**Weighted cohort**				**Generalized weighted cohort**		
β	Scenario	N	$\bar{N}$	% cens	reBias	MSE	Coverage	reBias	MSE	Coverage	Invalid weights	reBias	MSE	Coverage
β=0	A1	250	103	51	0.001	0.004	0.946	0.002	0.004	0.949	0.000	0.002	0.005	0.947
		500	205		0.001	0.002	0.953	0.001	0.002	0.943	0.000	0.001	0.002	0.946
		750	307		−0.001	0.001	0.943	−0.001	0.001	0.940	0.000	−0.001	0.002	0.951
	A2	250	47	33	−0.001	0.007	0.935	<0.001	0.011	0.919	0.053	<0.001	0.010	0.925
		500	94		0.002	0.003	0.940	0.001	0.005	0.934	0.001	0.002	0.005	0.937
		750	141		−0.003	0.002	0.930	−0.003	0.003	0.928	0.000	−0.003	0.003	0.930
	A3	250	48	33	0.001	0.017	0.937	−0.003	0.045	0.930	0.212	0.002	0.063	0.894
		500	95		<0.001	0.007	0.956	−0.003	0.017	0.940	0.102	−0.004	0.026	0.918
		750	143		0.002	0.005	0.951	0.003	0.011	0.936	0.040	0.004	0.015	0.928
β=0.3	A1	250	105	51	−0.045	0.004	0.939	−0.100	0.005	0.918	0.000	−0.051	0.005	0.944
		500	209		−0.040	0.002	0.940	−0.102	0.003	0.895	0.000	−0.045	0.002	0.940
		750	313		−0.050	0.002	0.920	−0.113	0.003	0.832	0.000	−0.058	0.002	0.925
	A2	250	48	33	−0.247	0.012	0.816	−0.106	0.011	0.927	0.050	−0.146	0.012	0.897
		500	97		−0.244	0.009	0.727	−0.119	0.006	0.912	0.002	−0.146	0.007	0.897
		750	145		−0.256	0.008	0.602	−0.129	0.005	0.878	0.000	−0.159	0.006	0.849
	A3	250	49	32	−0.291	0.025	0.868	0.004	0.048	0.909	0.199	0.015	0.065	0.892
		500	97		−0.264	0.014	0.850	−0.040	0.017	0.937	0.082	−0.033	0.026	0.930
		750	146		−0.246	0.010	0.808	−0.039	0.011	0.936	0.027	−0.013	0.015	0.942
β=1	A1	250	121	50	−0.076	0.010	0.744	−0.044	0.008	0.908	0.000	−0.004	0.007	0.942
		500	243		−0.075	0.008	0.590	−0.045	0.005	0.879	0.000	−0.005	0.003	0.951
		750	364		−0.076	0.007	0.425	−0.047	0.004	0.807	0.000	−0.007	0.002	0.953
	A2	250	58	33	−0.220	0.055	0.231	−0.048	0.013	0.909	0.012	−0.072	0.017	0.864
		500	117		−0.212	0.048	0.048	−0.051	0.008	0.897	0.000	−0.070	0.010	0.832
		750	175		−0.215	0.049	0.006	−0.054	0.006	0.843	0.000	−0.074	0.009	0.749
	A3	250	59	31	−0.332	0.125	0.197	0.010	0.040	0.899	0.097	0.041	0.065	0.877
		500	117		−0.313	0.105	0.043	−0.002	0.020	0.925	0.027	0.031	0.030	0.910
		750	176		−0.300	0.095	0.010	−0.004	0.012	0.939	0.004	0.036	0.020	0.916

**FIGURE 2:**
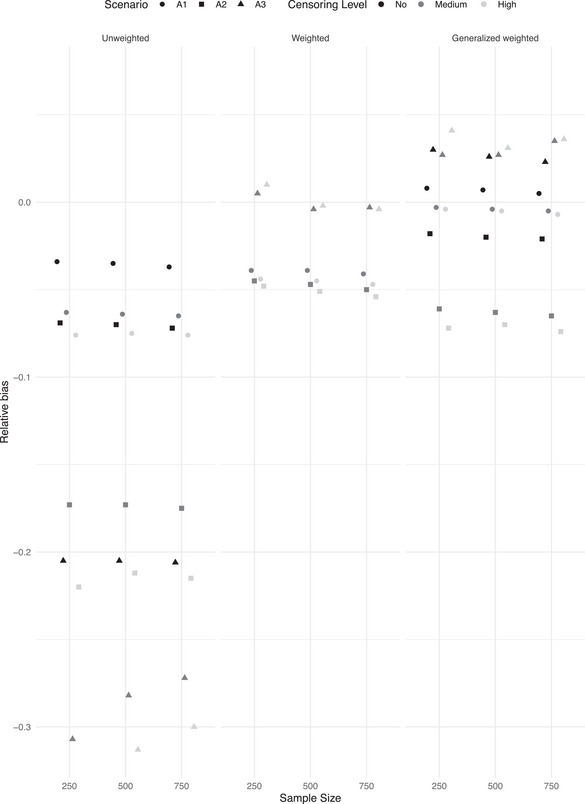
Simulation I. Relative bias (reBias) for $\hat{\beta }=1$ along 1000 trials for different levels of random right‐censoring, ascertainment, and sample sizes. A1: mild level of ascertainment; A2: medium level of ascertainment; A3: strong level of ascertainment.

If the covariate effect is strong (β=1), the naive unweighted method is outperformed by the weighted approaches, even when the level of outcome‐dependent selection is low (scenario A1). When the covariate effect is weaker (β=0.3), and the level of ascertainment is medium (scenario A2) or high (scenario A3), both weighted cohort methods perform similarly and clearly outperform the naive, unweighted approach. Under a weak level of ascertainment (scenario A1) and weak covariate effect, the new generalized weighted cohort method performs as well as the naive unweighted approach and they slightly outperform the traditional weighted cohort approach which presents a larger level of relative bias. Importantly, the traditional weighted approach is often not applicable when the assumed covariate effect is weak. Negative weights are obtained due to violation of condition (10). The same problem is observed in the null case scenario when assuming β=0. The new generalized weighted cohort method does not suffer from this problem, yielding valid results in all studied scenarios. When considering the impact of the level of random right‐censoring on the results, it is important to note that even if the naive unweighted Cox regression approach is in general outperformed by the weighted approaches, a systematic negative bias is observed in both the traditional and new generalized weighted cohort methods in most scenarios with medium and high‐level censoring. This is a consequence of the violation of the discrete right‐censoring assumption. As expected, the generalized weighted cohort method leads to unbiased results when random censoring is not present (Table 1). This cannot be evaluated with the traditional weighted cohort method since, as previously discussed, it provides invalid weights without censoring. Accordingly, as expected, bias increases with censoring levels for both weighted cohort methods, but the increase is not dramatic. A point of attention of both weighted cohort approaches is the observed slight overestimation of the covariate effect when samples are small (below 100 included families), in combination with high levels of censoring and strong outcome‐dependent sampling. This behavior tends to decrease when increasing the number of families included in the sample. Regarding the coverage probabilities, methods exhibit closeness to the nominal level of 0.95 when bias is negligible (e.g., generalized weighted cohort in the no random censoring scenario). Both weighted cohort methods show undercoverage of the 95% confidence intervals in the presence of moderate and high levels of right‐censoring in the scenarios with a nonnull covariate effect, attributable to the nonnegligible underestimation of the covariate effect.

#### 3.3.2 Simulation II

Table 4 shows the results assuming the presence of unobserved family‐shared heterogeneity. When unmeasured within‐family correlation is mild (θ=0.1), we found similar results as in the previous simulation setting: weighted methods perform similarly and provide better results than the unweighted Cox model. Also, weighted methods, which deal with outcome‐dependent sampling but ignore the presence of unobserved heterogeneity, perform better than a gamma shared frailty model which deals with shared unobserved heterogeneity but ignores outcome‐dependent sampling.

**TABLE 4:** Simulation II. Relative bias (reBias), mean square error (MSE), and coverage probability (Coverage) for $\hat{\beta }$ along 1000 trials. A1: mild level of ascertainment; A2: medium level of ascertainment; A3: strong level of ascertainment; N: number of families. Data are generated according to a shared frailty model with frailty variance θ. For the weighted approaches, the proportion of invalid (negative) weights along 1000 trials is also reported.

				**Unweighted**			**Weighted cohort**				**Generalized weighted cohort**			**Shared frailty**		
θ	β	Scenario	N	reBias	MSE	Coverage	reBias	MSE	Coverage	Invalid weights	reBias	MSE	Coverage	reBias	MSE	Coverage
θ=0.1	β=0	A1	500	0.002	0.002	0.953	0.002	0.002	0.953	0.000	0.003	0.002	0.947	<0.001	0.002	0.951
		A2	500	0.002	0.002	0.953	0.002	0.003	0.946	0.000	0.003	0.003	0.953	0.002	0.002	0.961
		A3	500	−0.001	0.006	0.949	<0.001	0.013	0.939	0.060	0.001	0.015	0.938	−0.001	0.006	0.957
	β=0.3	A1	500	−0.062	0.002	0.912	−0.092	0.002	0.896	0.000	−0.060	0.002	0.929	−0.062	0.002	0.923
		A2	500	−0.202	0.006	0.728	−0.078	0.003	0.929	0.000	−0.119	0.004	0.898	−0.202	0.006	0.747
		A3	500	−0.251	0.012	0.821	−0.042	0.014	0.947	0.049	−0.039	0.016	0.934	−0.251	0.012	0.836
	β=1	A1	500	−0.090	0.010	0.417	−0.058	0.006	0.764	0.000	−0.032	0.003	0.901	−0.090	0.010	0.425
		A2	500	−0.195	0.040	0.027	−0.055	0.007	0.853	0.000	−0.080	0.010	0.730	−0.194	0.040	0.024
		A3	500	−0.295	0.093	0.034	−0.015	0.017	0.917	0.011	0.002	0.023	0.909	−0.295	0.093	0.046
θ=1	β=0	A1	500	−0.001	0.002	0.960	−0.001	0.002	0.955	0.000	−0.001	0.002	0.951	−0.001	0.002	0.970
		A2	500	−0.001	0.002	0.952	<0.001	0.005	0.954	0.000	−0.001	0.004	0.949	−0.001	0.002	0.957
		A3	500	−0.001	0.007	0.943	−0.002	0.022	0.922	0.071	−0.006	0.024	0.925	−0.001	0.007	0.952
	β=0.3	A1	500	−0.186	0.005	0.722	−0.118	0.004	0.894	0.000	−0.137	0.004	0.877	−0.134	0.003	0.856
		A2	500	−0.252	0.008	0.603	0.031	0.006	0.939	0.000	−0.072	0.005	0.923	−0.243	0.008	0.640
		A3	500	−0.299	0.015	0.768	−0.008	0.022	0.923	0.055	−0.095	0.024	0.915	−0.302	0.015	0.775
	β=1	A1	500	−0.209	0.046	0.006	−0.133	0.021	0.370	0.000	−0.144	0.024	0.336	−0.117	0.016	0.303
		A2	500	−0.264	0.072	0.002	−0.034	0.007	0.929	0.000	−0.010	0.016	0.710	−0.210	0.048	0.048
		A3	500	−0.340	0.123	0.030	−0.039	0.030	0.872	0.015	−0.098	0.041	0.859	−0.340	0.123	0.032

For strong within‐family correlation (θ=1), the performance of both weighted methods is, in general, good if the level of ascertainment is moderate or high (scenarios A2 and A3). If the level of ascertainment is mild (scenario A1), weighted methods would still outperform the traditional unweighted Cox approach but a shared frailty model seems a better choice in this setting. Bias is still noticeable with the shared frailty model, but of a smaller magnitude. Finally, the original weighted cohort also provided negative weights in this setting with unobserved family‐shared heterogeneity, while the newly proposed generalized weighted cohort method proved to be more robust.

Overall, the simulation results show that the new generalized weighted cohort method performs similarly to the original weighted cohort approach proposed by Antoniou et al. (2005) but it is more generally applicable. The original weighted cohort method performs well, in general, in the presence of a combination of a strong covariate effect and strong outcome‐dependent sampling, as expected. However, its applicability is not general enough, leading invalid weights in relevant practical situations, especially when covariate effect is weak. The performance of the new generalized weighted cohort approach is in generally satisfactory, a point of attention is the observed slight overestimation of the covariate effect with very small samples and large levels of censoring in combination with strong levels of outcome‐dependent sampling. Our sensitivity analysis, based on assuming the existence of unobserved heterogeneity, shows that inverse probability of selection weighted Cox models can still perform properly in the presence of mild unobserved family‐shared heterogeneity, but they lead to biased results when the size of the frailty variance is large. Still, weighted methods seem to be preferred over the alternative approach of ignoring outcome‐dependent sampling and fitting a shared frailty model if the level of outcome‐dependent sampling is strong. If the level of ascertainment is mild, the results indicate a preference for the shared frailty model.

### 3.4 Software Implementation

The generalized weighted cohort method developed in this work was implemented in the user‐friendly R package “wcox,” which can be downloaded from https://github.com/vharntzen/wcox. Moreover, all code used in the Simulation study is available as the Supporting Information.

## 4 Real Data Applications

We present two applications to illustrate the performance of the new generalized weighted cohort method compared to the traditional approaches on real data. In both applications, the goal is to assess the association between common susceptibility *loci* (gene locations on the chromosome) identified in GWAS and cancer, using data collected through genetic testing in clinical genetics units. Specifically, the first application is devoted to study the association between a single nucleotide polymorphism (SNP) and CRC in carriers of a pathogenic variant in the *PMS2* gene while the second one focuses on the association of a 161 SNP‐based PRS with breast cancer. The selection of both data sets was based on family history of cancer with oversampling of cancer cases with the aim of finding carriers of certain genetic variants. As a result, the sample used in the first application is composed of *PMS2* mutation carriers. In the second application, the sample is composed of women with a family history of breast cancer and without *BRCA1* or *BRCA2* mutations.

### 4.1 Application to CRC

**FIGURE 3:**
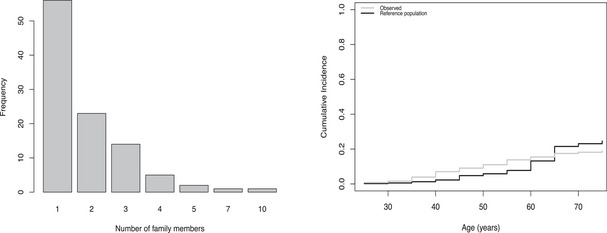
Application 1: Study of the association between SNP rs1321311 and CRC cancer in male carriers of a pathogenic variant in the gene *PMS2*. Left panel: Size of the families included in the sample. Right panel: Cumulative incidence of colorectal cancer at different ages. The gray line shows the observed risk in the sample. The black line reflects the expected cumulative colorectal cancer risk for the population of *PMS2* mutation carriers based on previous literature (ten Broeke et al. 2015). Specifically, age‐specific CRC incidence rates of *PMS2* mutation carriers are obtained multiplying the point estimates of the age‐dependent hazard ratios as reported in Table 2 in ten Broeke et al. (2015) by the underlying population‐based incidence rates of CRC for males in the Netherlands in 2011 according to the Netherlands Cancer Registry (NCR).

In this application, we consider a sample of male carriers of the germline *PMS2* mutation. Motivated by the previous promising findings reported by ten Broeke et al. (2018), we studied the association between the SNP rs1321311 and CRC in men. The sample consisted of 191 males belonging to 102 different families collected in eight Dutch clinical genetics centers between 2007 and 2016. Details on the selection criteria can be found in ten Broeke et al. (2015). The distribution of the number of individuals belonging to the same family was very skewed, the mean number of individuals per family was 1.83 and most of the families (55 %) contributed with one single member (Figure 3, left panel). The last age of follow‐up ranged between 25 and 88 years, but given that no events were observed after 75 years old, we censored observations at 75 years. The range of observed ages at CRC diagnosis varied between 25 and 75, and 58 events were observed (70% censoring). From the 191 studied individuals, 116 were homozygotes of the nonrisk allele, 65 were heterozygotes, and 10 were homozygotes of the risk allele. Because of the limited size of the last category, we evaluated the effect of the indicator of being a carrier of the rs1321311 allele.

We considered four different models: unweighted Cox regression, the state‐of‐the‐art weighted cohort method, our new generalized weighted cohort method based on the new and more general weighting scheme, and a shared gamma frailty model as a sensitivity analysis to measure the potential impact of unobserved family‐specific heterogeneity. The two studied weighted methods require the knowledge of incidence rates for CRC in carriers of pathogenic variants in *PMS2*. These were obtained by multiplying the population‐based incidence rates of CRC in the Netherlands in 2011 (NCR 2021) by the previously published (ten Broeke et al. 2015) age‐dependent hazard ratios of CRC for *PMS2* carriers. The choice of the year 2011 as the reference is justified because it is the middle point of the data collection period (2007–2016). The specific age‐specific intervals and incidence rates used in this application can be found in Table S2. As in the simulation study, robust standard errors were obtained using the Huber–White sandwich estimator for the weighted approaches while the usual normal approximation was used for the unweighted Cox and frailty models.

From the results reported in the bottom line of Table 5, it is observed that the new generalized weighted cohort method provides slightly larger estimated effects than the well‐known (unweighted) Cox regression. In agreement to the result obtained with the unweighted method, the estimated association between the risk allele rs1321311 and CRC was statistically significant at the usual 5% level when using the generalized weighted cohort method. Importantly, the traditional weighted cohort approach could not be used because negative weights were obtained. Specifically, the oversampling of cases was not strong enough in the age group 65–70 years old, and restriction (10) discussed in Section 2.1 was not met leading to negative weights for unaffected individuals in this age group. The shared frailty model provides the lower estimated covariate effect among the evaluated methods. This is probably due to the limited sizes of the family clusters and a small underlying unobserved heterogeneity. The estimated frailty variance was 0.15 with a broad confidence interval (0,1), indicating difficulties of the model to give reliable estimates of the level of unobserved heterogeneity. A likely major driving cause for this difficulty is the limited cluster size of this application since most of the families contribute a single individual to the analysis. As a consequence, the shared frailty approach is not recommended in this application and one would rather choose for the new generalized weighted cohort approach.

**TABLE 5:** Application to CRC in male carriers of *PMS2*. Estimated regression coefficients ($\hat{\beta }$) and corresponding 95% confidence intervals for the effect of the SNP rs1321311 for different Cox models. Case weights are calculated based on incidence rates of CRC for *PMS2* mutation carriers defined as the point estimates of the age‐dependent hazard ratios reported in ten Broeke et al. (2018) multiplied by the population‐based rates of CRC in the Netherlands in 2011.

Model	$\hat{\beta }$ (95% CI)
Unweighted	0.723 (0.182–1.265)
Frailty	0.671 (0.149–1.192)
Weighted cohort	− (*negative weights*)
Generalized weighted cohort	0.771 (0.234–1.308)

### 4.2 Application to Breast Cancer

**FIGURE 4:**
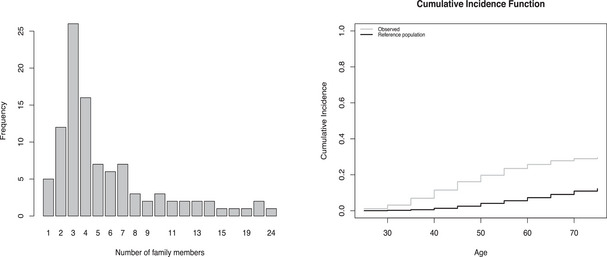
Application 2: Study of the association between a polygenic risk score and female breast cancer. Left panel: Size of the families included in the sample. Right panel: Cumulative incidence of breast cancer at different ages. The gray line shows the observed risk in the sample. The black line shows the population‐based (the Netherlands, 2001 (NCR 2021)) cumulative incidence used as reference in the weighted analyses.

In this application, the association between a PRS score and breast cancer was analyzed using a sample of 579 clinically ascertained women belonging to 101 families. On average, six women were included per family (mean family size = 5.73 and standard deviation = 4.66, Figure 4 left panel). The inclusion criterion was twofold. Per family, one of the women should be tested negative for *BRCA1* or *BRCA2* pathogenic variants. This was a special feature of this sample and means that family aggregation and early onset of cancer are not explained by pathogenic variants in these high‐risk genes. Furthermore, breast cancer had to occur in at least three female family members or in two females if at least one had bilateral breast cancer before the age of 60. The families were selected between 1990 and 2012 by Clinical Genetic Services in four Dutch cities (Groningen, Leiden, Nijmegen, and Rotterdam) and one Hungarian city (Budapest). Given the scarcity of events after 80 years of age (only one observed event at 90), we censored observations at age 80, resulting in 322 observed events (44% censoring).

The PRS was based on 161 SNPs weighted by previously published log‐odds ratios (mostly based on population‐based case–control studies). Detailed description of the calculation of PRS can be found elsewhere (Lakeman et al. 2019). As before, to establish the association between the marker of interest, the PRS, and breast cancer, we considered four different models: the traditional unweighted Cox regression, the state‐of‐the‐art weighted cohort method to deal with outcome‐dependent sampling, our new weighted method, and a shared gamma frailty model and we used the same approach as before for the calculation of standard errors. Population‐based incidence rates of the Netherlands in 2001 (NCR 2021) (midpoint of the sample selection period) were used as external input to construct the weights. The specific age‐specific intervals and incidence rates used in this application can be found in Table S2.

From the results reported in Table 6, we observe that the new generalized weighted cohort method provides a slightly smaller effect than the previously proposed weighted cohort approach and that both provided smaller effects than the unweighted Cox model. None of these three approaches reached statistical significance at the 5% level. In order to estimate the level of heterogeneity due to unmeasured within‐family similarity, a shared frailty model was also fitted. The estimated frailty variance was 0.41, indicating that unobserved heterogeneity is not negligible in this application. This, together with the large size of the included families, is probably the reason why the shared frailty model provides larger estimates of the conditional log‐hazard ratio than the log‐hazard ratio provided by the other methods, even if statistical significance at the 5% level is also not reached with this approach. According to our simulation results, we hypothesize that the association between PRS and breast cancer is likely obscured by ignoring the strong unobserved heterogeneity and that the frailty approach is preferred in this application.

**TABLE 6:** Application to female breast cancer in non‐*BRCA1*/*2* families. Estimated regression coefficients ($\hat{\beta }$) and corresponding 95% confidence intervals for the effect of polygenic risk score (PRS) for different Cox models.

Model	$\hat{\beta }$ (95% CI)
Unweighted	0.110 (−0.096,0.317)
Frailty	0.173 (−0.045, 0.390)
Weighted cohort	0.079 (−0.226, 0.385)
Generalized weighted cohort	0.062 (−0.261, 0.384)

The two real data applications offer interesting complementary insights and enable us to formulate recommendations for practical analysis of real data. We suggest conducting an analysis using a weighted cohort approach alongside a frailty model analysis as a sensitivity check. If the estimated frailty variance is substantial, significantly deviating from zero, one would anticipate that the weighted approach (either traditional or generalized) underestimates the covariate effect. Conversely, if the estimated frailty variance is very close to zero, attention should be directed solely to the results obtained from the weighted cohort analysis. Both the traditional and generalized weighted cohort methods are expected to yield comparable results; however, the new generalized method is assured to provide valid weights, whereas this is not guaranteed for the traditional method.

## 5 Discussion

In this paper, we have revisited the analysis of outcome‐dependently sampled survival data with weighted Cox regression using external data to construct inverse probability of selection weights. Our research is motivated by the interest in the effect of potential modifying factors on cancer risk using clinically ascertained data. Typically, those data sets are collected through ongoing genetic testing programs, where selection criteria lead to an overrepresentation of young cases and hence, the resulting samples are not representative of the target population of interest. We have introduced a new weighting scheme that restores the expected ratio of events and nonevents at each follow‐up time using population‐based hazard information.

A strength of the new weighting scheme is that it relies on fewer assumptions to provide valid, nonnegative weights. The traditional weighted cohort (Antoniou et al. 2005) approach requires that a number of conditions are fulfilled, which hamper its applicability. Specifically, the original method is problematic if oversampling of cases is not observed in all age groups. In practice, although overall oversampling of events is expected, it does not necessarily hold for all age groups. Our new method overcomes this restriction and can be applied to a wider set of oversampling schemes, hence it can be regarded as a generalization of the traditional weighted cohort approach. Our simulation study and real data applications have practically shown that the new generalized weighted cohort method can be applied in a broader set of situations than the original weighted cohort. This together with user‐friendly implementation makes it an attractive analysis tool for applied researchers in the field.

Similarly to the previously proposed weighted cohort method, our approach relies on a number of assumptions. Our work has contributed to clarifying and studying in‐depth the intrinsic assumptions of weighted Cox regression approaches based on external data to address outcome‐dependent sampling. This has helped us gain a better understanding of the merits and limitations of this attractive type of approach due to its simplicity. First, a crucial assumption is the existence of a well‐established external source of population‐based incidence rates. Second, the sampling probabilities of observed individuals depend on the age at onset but they are assumed to be conditionally independent of the risk modifier under investigation. These two assumptions have been previously discussed in the context of the weighted cohort method (Antoniou et al. 2005; Barnes et al. 2012). Another assumption, not previously discussed but common to both the original and generalized weighted cohort methods, is discrete right‐censoring, which means that random censoring within intervals cannot be handled. Our simulation study has shown that this assumption typically leads to underestimation of effects and seems to be a limitation difficult to overcome in absence of extra information. In addition, both approaches currently assume nontruncation and time‐fixed covariates. Extension of the current generalized weighted cohort approach to properly deal with these phenomena is left as future research. Furthermore, the relationship between the hazard and the risk modifier under investigation should approximately follow a proportional hazards specification. We have examined the performance of both the traditional and the new generalized weighted cohort approaches under model misspecification, specifically, under noncollapsability due to the presence of residual familial aggregation. In this case, we have also observed that the use of weighted approaches seems advisable compared to the naive unweighted approach. In addition, if the number of available individuals per family is limited, which is the most common situation in practice, the new generalized weighted cohort method might be the preferred option, outperforming a shared frailty model and the traditional weighted cohort approach. However, we would like to caution about the interpretation of the estimated effect and point out the systematic downward bias of the regression coefficient in this setting, proposing the systematic inclusion of the results of a shared frailty model as a sensitivity analysis.

The extension of weighting approaches to deal with outcome‐dependent sampling to the context of frailty models would be interesting but challenging. Since the estimated incidence rate in the sample depends on the correct estimation of the frailty variance, it would be necessary to know the value of the frailty variance to derive correct weights. However, the frailty variance is latent and hence we anticipate an identifiability problem in such an approach. More sophisticated modeling, using a frailty model with explicit correction for ascertainment is possible but not straightforward and it is left as future research. It is noteworthy that such a complex approach will presumably require large clusters and sample sizes and hence our simpler approach based on borrowing information from a trustworthy external source will still be preferred in a number of relevant practical situations, such as our application to *PMS2* carriers. Moreover, this paper did not specifically address the determination of standard errors for the new and existing weighted cohort approaches, a topic that would require further investigation. As pointed out in previous studies (Seaman and White 2013), the widely used sandwich adjustment method may show an anticonservative behavior as it overlooks the variability in weight estimation. It would be advisable to investigate alternative methodologies within the sandwich principle framework.

In conclusion, for performing regression analysis using survival data obtained under family‐based outcome dependently sampling, specialized techniques are required to avoid bias and provide valid inference. Inverse probability of selection weighted Cox models based on external data are feasible and simple approaches leading to reasonable results in many realistic scenarios, particularly when the level of clinical ascertainment is strong. We have proposed a more general method than existing ones, which has a broad range of applicability. Given the demonstrated tendency for weighted cohort approaches to underestimate covariate effects in the presence of unobserved heterogeneity, we recommend always incorporating a sensitivity analysis based on a frailty model. This analysis serves to evaluate the degree of familial clustering in the data set and to assess the expected level of underestimation by the weighted cohort methods.

## Conflicts of Interest

The authors declare no conflicts of interest.

## Open Research Badges

This article has earned an Open Data badge for making publicly available the digitally‐shareable data necessary to reproduce the reported results. The data is available in the Supporting Information section.

This article has earned an open data badge “**Reproducible Research**” for making publicly available the code necessary to reproduce the reported results. The results reported in this article could fully be reproduced.

## Supporting information

Supporting Information

## Data Availability

The data that support the findings of this study are available from the corresponding author upon reasonable request.

## Appendix A

### A.1 Unbiasedness of the Inverse Probability of Selection Weighted Cox Approach and Large Sample Properties

#### A.1.1 Unbiasedness of the Inverse Probability of Selection Weighted Cox Approach

Denote by D the selection event, and let W(t) be the probability of being selected given that the observed event time is t, assume that W(t)>0 on the support of T. Our approach to show unbiasedness follows the steps proposed by Mandel et al. (2018, section 2.1.) to show unbiasedness in the context of a Cox model for random double‐truncated data. Given the assumption D⊥T|Z, our outcome‐dependent sampling mechanism can be conceptualized as a right‐truncation setting where the distribution about the right‐truncation time is not explicit, but it can be inferred from external data.

In the absence of covariates, the density of the observed data, obtained under biased (in particular within an outcome‐dependent sampling where D⊥Z|T) is given by the following weighted density:

$$f_{T|D}\left(t\right)=\frac{P\left(D=1|T=t\right)f_{T}\left(t\right)}{\int_{0}^{\infty }P\left(D=1|T=s\right)f_{T}\left(s\right)ds}.$$

Accordingly, the joint density of the sampled information including covariate Z is given by

$$f_{T,Z|D}\left(t,z\right)=\frac{W\left(t\right)h\left(t|z;\beta \right)\text{exp}\left(-\int_{0}^{t}h\left(y|z;\beta \right)dy\right)f_{Z}\left(z\right)}{E(W(T))}.$$

The density of the event times conditional on the covariate is therefore

$$f_{T|Z,D}\left(t,z\right)=\frac{W\left(t\right)h\left(t|z;\beta \right)\text{exp}\left(-\int_{0}^{t}h\left(y|z;\beta \right)dy\right)}{E(W(T)|z)}.$$

Note that E(W(T)|z)=P(D=1|T,z) depends on β and thus this biased sampling must be accounted for in the estimation of β. However, since the weight W(t) is a function of t alone, based on the key assumption D⊥Z|T, it follows that $f_{Z|T,D}=f_{Z|T}$ and the following standard probabilistic result from the Cox model can be used:

$$E\left(Z|T=t,D\right)=E\left(Z|T=t\right)=\frac{E\left[Ze^{\beta Z}{\bar{F}}_{T|Z}\left(t|Z\right)\right]}{E\left[e^{\beta Z}{\bar{F}}_{T|Z}\left(t|Z\right)\right]},$$

where $\bar{F}=1-F$ denotes the survival function.

Let $f_{Z|D}=E\left(W\left(T\right)|z\right)f_{Z}\left(z\right)/E\left(W\left(T\right)\right)$ denote the marginal weighted density of the covariate, then we can adapt the former general result to our setting and rewrite it as follows:

$$E\left(Z|T=t,D\right)=\frac{E\left[Ze^{\beta Z}{\bar{F}}_{T|Z}\left(t|Z\right)/E\left(W\left(T\right)|Z\right)|D\right]}{E\left[e^{\beta Z}{\bar{F}}_{T|Z}\left(t|Z\right)/E\left(W\left(T\right)|Z\right)|D\right]}.$$

The former expression still involved functionals of Z and T unconditionally on D. To rewrite the expectation as a function of observed variables, we use the expression on the density of the event times conditional on the covariate $f_{T|Z,D}\left(t,z\right)$ and the fact that W(t)>0 on the support of T, which implies ${\bar{F}}_{T|Z}\left(t|z\right)/E\left(W\left(T\right)|Z=z\right)=E\left[W{\left(T\right)}^{-1}I\left(T\geq t\right)\left|Z=z,D\right)\right]$. As a result

$$E\left(Z|T=t,D\right)=\frac{E\left[Ze^{\beta Z}W{\left(T\right)}^{-1}I\left(T\geq t\right)|D\right]}{E\left[e^{\beta Z}W{\left(T\right)}^{-1}I\left(T\geq t\right)|D\right]},$$

which implies the unbiasedness of the weighted estimating equation

$$U\left(\beta \right)=\sum_{i=1}^{n}\{Z_{i}-\frac{\sum_{j-1}^{n}Z_{j}e^{\beta Z_{j}}(W{\left(T_{j}\right)}^{-1}I\left(T_{j}\geq T_{i}\right)}{\sum_{j-1}^{n}e^{\beta Z_{j}}(W{\left(T_{j}\right)}^{-1}I\left(T_{j}\geq T_{i}\right)}\}.$$

#### A.1.2 Large Sample Properties

We consider independent realizations $\left(T_{1},Z_{1}\right)$, …, $\left(T_{n},Z_{n}\right)$ having the law of (T,Z)|D. The following regularity conditions are assumed:

1. T is continuous and $t_{min}<T<t_{max}$ with probability one for some $t_{min}\geq 0$ and $t_{max}\leq \infty$.
2. The covariate Z is bounded.
3. W(t)>ε and $\hat{W}\left(t\right)>\varepsilon$ on $\left[t_{min},t_{max}\right]$ for some ε>0.
4. The limiting second derivative of PL(β) is positive definite, where
  PL(β)=1n∑i=1n(βt−β0t)Zilog1n∑j=1neβtZj{W^(Tj)}−1I{Tj≥Ti}1n∑j=1neβ0tZj{W^(Tj)}−1I{Tj≥Ti}−log1n∑j=1neβtZj{W^(Tj)}−1I{Tj≥Ti}1n∑j=1neβ0tZj{W^(Tj)}−1I{Tj≥Ti}
  is the centered pseudo‐log likelihood that vanishes at $\beta_{0}$.

Moreover, the following two conjectures about $\hat{W}$ are assumed:

C1. $\max_{1\leq i\leq n}\left\{\hat{W}\left(T_{i}\right)-W\left(T_{i}\right)\right\}\to 0$ in probability (uniform convergence).
C2. $\sqrt{n}\left\{\hat{W}\left(t\right)-W\left(t\right)\right\}=n^{-1/2}\sum_{i=1}^{n}\zeta_{n}\left(D_{i},t\right)+o_{p}\left(1\right)$ uniformly on $t\in [t_{min},t_{max}]$, where $D_{i}=\left(T_{i},Z_{i}\right)$ is the data for subject i, and $\zeta_{n}\left(D_{i},t\right)$ are independent and identically distributed zero mean random variables having finite variance.

Then, under the regularity conditions 1–4 and conditions C1 and C2, the solution of expression (5) in the main text with $\hat{W}$ plugged‐in is consistent and asymptotically normal. The proof can be found in supplementary material A of Mandel et al. (2018). For proof of conjectures C1 and C2, we refer to de Uña Álvarez and Van Keilegom (2021).

### A.2 Literature Review: Family Size and the Use of the Weighted Cohort Approach

Among the 81 citations of Antoniou et al. (2005)'s paper, we found 51 papers that used a weighted cohort approach to obtain unbiased Hazard Ratios in a Cox model (list available upon request). The majority (62.7%, n = 32) were studies into breast and ovarian cancer risks. In 48 of the 51 papers applying the weighted cohort approach, the study sample certainly includes multiple members per family, but the exact number of families was only mentioned in 19 papers, shown in Table A1. For each study, we calculated the average number of family members included. Note that this may fluctuate: one study (Andrieuet al. 2006) described the exact family composition of the sample (see Table A1 footnote f). The median of the paper‐specific, average family cluster sizes was 2.5. Generally, these data were collected at family cancer‐ or genetics clinics, where relatives of the index case (proband) were invited to be tested. Sometimes this was combined with “population‐based” recruitment (Ouakrim et al. 2015; Chau et al. 2016).

**TABLE A1:** Family information (when reported) in papers applying weighted cohort approach. This list is a subset of all (81) PubMed citations of the paper of Antoniou et al. (2005) on June 2, 2021, with inclusion criteria (1) applying weighted Cox, (2) mentioning the number of families and sample size.

***Authors***	**Year**	**Sample**	**Average number of relatives per family**	**(Cancer) research area**
Borde et al.	2021	578 families; 760 carriers	1.6	Breast
Dashti et al.	2018	774 families; 2042 carriers	2.6	Colorectal
ten Broeke et al.	2018	152 families; 521 samples	3.4	Colorectal
Kamiza et al.	2016	62 families; 260 carriers	4.2	Colorectal
Dashti et al.	2017	761 families; 1925 carriers	2.5	Colorectal
Chau et al.	2016	15,049 families; 42,489 participants^a^	5.3	Colorectal
Win et al.	2015	330 families; 1098 carriers	3.3	Colorectal
Win et al.	2015	593 families; 854 individuals	1.4	Colorectal
Dashti et al.	2015	548 families; 1128 women	2.1	Endometrial
Ouakrim et al.	2015	748 families; 1858 carriers^b^	2.5	Colorectal
Pooley et al.	2014	3134 families; 4822 included^c^	1.5	Breast
Killick et al.	2014	115 families; 158 included^d^	1.4	Breast
Win et al.	2013	315 families; 927 carriers	2.9	Colorectal
Pijpe et al.	2012	930 families; 1122 carriers^e^	1.2	Breast
Win et al.	2011	498 families provided 1324 carriers; 287 families provided 1219 noncarriers	2.7(carriers); 4.2 (noncarriers)	Colorectal
Win et al.	2011	286 families provided 601 carriers; 182 families provided 533 noncarriers	2.1(carriers); 2.9 (noncarriers)	Endometrial
Amos et al.	2010	93 families; 489 included (of which 45 married‐in)	5.3	Lung
Antoniou et al.	2006	392 families; 810 carriers	2.1	Breast
Andrieu et al.	2006	1074 families; 1601 women	1.5^f^	Breast

^a^ Includes some population‐based recruitment for which average number of recruited relatives per family was 2.6, versus 5.3 for clinic‐based families.

^b^ 25% population‐based.

^c^ Sampled noncarrying relatives only.

^d^ Relatives functioned as controls, that is, noncarriers.

^e^ This concerns only one of the data sets in the paper.Pijpe

^f^ The relative occurrence of relatives per family was 71.1% for size 1, 17.9% for size 2, 6.2% for size 3, 2.8% for size 4, and 2.0% for size 5 up until 11.

### A.3 Population Incidence Rates Used in Real Data Applications (A2)

**TABLE A2:** Population‐based age‐specific incidence rates (in cases per 100.000) used for weight construction. For breast cancer, we used the registered data of The Netherlands in 2001 for women (NCR 2021). For colorectal cancer, age‐specific incidence rates were obtained by multiplying the population‐based incidence rates of CRC in the Netherlands in 2011 (NCR 2021) by the previously published (ten Broeke et al. 2015) age‐dependent hazard ratios of CRC for *PMS2* carriers. The choice of the year 2011 as the reference is justified because it is the middle point of the data collection period (2007–2016).

Age group	Colorectal (men)	Breast (women)
25–30	40.56	9.09
30–34	69.39	33.61
35–39	146.60	72.41
40–44	204.38	156.17
45–49	518.28	241.48
50–54	221.10	323.56
55–59	411.68	310.83
60–64	1214.11	363.63
65–69	2016.21	388.41
70–74	405.12	415.42
75–79	—	293.22
