# Supplementary material for: A New Inverse Probability of Selection Weighted Cox Model to Deal With Outcome‐Dependent Sampling in Survival Analysis
Source: Biom J. 2025 Jun 11;67(3):e70056. doi: 10.1002/bimj.70056 (PMC12159397; doi:10.1002/bimj.70056)
Supplement: Supplementary file 1 — Supporting Information [file BIMJ-67-e70056-s001.zip › Code for Biometrical Journal ThirdCheck/Synthetic Data Analysis/SyntheticBreastCancerDataAnalysis.html]

Analysis of synthetic breast cancer dataset


# Analysis of synthetic breast cancer dataset

#### 2024-10-26

# Introduction

This document presents the analysis of a synthetic data based on real
breast cancer data. The synthetic dataset was obtained using the
function syn from synthpop R package; using parametric methods
(method=“parametric”).

# Load dataset and required functions for analysis

```
# Set the working directory to the 'Synthetic Data Analysis' folder, load dataset, and  source required scripts for weight calculation

#setwd("Synthetic Data Analysis")
data<-read.table("SyntheticDataBreast.txt",header=T,sep="") 
source("../Simulations/prepare_data_calculate_weights.R")
```

# Preprocessing and descriptive analysis of the dataset

```
data$idi<-1:nrow(data)# add individual indicator

#Censor events at 80 or older age due tue scarcity of events (in line to what is done in the analysis with the real data)
data$d[data$y>=80]<-0
data$y[data$y>=80]<-79
```

The synthetic dataset presents a similar level of censoring (45 %)
and family size distribution than the real breast cancer dataset.

```
barplot(table(table(data$family)),xlab="Number of family members",ylab="Frequency")
```

# Construction of weights

This analysis uses population 5-year incidence rates from the
Netherlands in 2001 as external data (source Netherlands
Cancer Institute

```
# Define age groups as intervals for analysis, with each interval representing a 5-year age range.
breaks = c(25,30,35,40,45,50,55,60,65,70,75,80)
# Set population incidence rates per age group (per 100,000 individuals) as a vector.
# These values represent cancer incidence rates for each age range defined in 'breaks'.
mu_k<-c(
9.09,
33.61,
72.41,
156.17,
241.48,
323.56,
310.83,
363.63,
388.41,
415.42,
293.22
)/100000
```

We will now calculate the weights using both the traditional weighted
cohort approach and the newly proposed generalized weighted cohort
method.

```
# Prepare the data for analysis by using the 'Prepare_data' function.
# This function takes the dataset 'data', population incidence rates 'mu_k', and age group breaks 'breaks'.
df<-Prepare_data(dat=data, population_incidence=mu_k, breaks=breaks)
# Calculate the newly proposed weights based on the prepared data frame 'df' using the #'calculate_weights_new' function.
df$vector_weights_new<-calculate_weights_new(df)
```

```
## [1] "No negative weights"
```

```
# Calculate weights according to the Antoniou method using the prepared data frame 'df'.
df$vector_weights_antoniou<-calculate_weights_antoniou(df)
```

```
#Display Antonious weights
# Step 1: Extract unique values of y_cat, d, and vector_weights_antoniou
summary_table <- df %>%
  distinct(y_cat, d, vector_weights_antoniou) %>%
  rename(weight = vector_weights_antoniou)

# Step 2: Reshape the summary table to separate columns for each value of d
summary_table_wide <- summary_table %>%
  pivot_wider(names_from = d, values_from = weight, names_prefix = "weight_d", values_fill = list(weight_d0 = 0, weight_d1 = 0))

# Step 3: Display the summary table with a title
summary_table_wide %>%
  kable(caption = "Unique Antoniou's Weights by Age Category and Censoring Status")
```

Unique Antoniou’s Weights by Age Category and Censoring
Status

| y\_cat | weight\_d1 | weight\_d0 |
| --- | --- | --- |
| (25,30] | NaN | NA |
| (30,35] | 0.0680799 | 4.9606604 |
| (35,40] | 0.0990087 | 2.6959836 |
| (40,45] | 0.1565125 | 2.9329922 |
| (45,50] | 0.2251415 | 2.8654002 |
| (50,55] | 0.4036741 | 1.8944889 |
| (55,60] | 0.4337976 | 1.6710547 |
| (60,65] | 0.6456542 | 1.2976505 |
| (65,70] | 0.5787257 | 1.5529225 |
| (70,75] | 0.7171893 | 1.2686702 |
| (75,80] | 1.8014779 | 0.8824499 |

```
#Display new  weights
# Step 1: Extract unique values of y_cat, d, and vector_weights_antoniou
summary_table <- df %>%
  distinct(y_cat, d, vector_weights_new) %>%
  rename(weight = vector_weights_new)

# Step 2: Reshape the summary table to separate columns for each value of d
summary_table_wide <- summary_table %>%
  pivot_wider(names_from = d, values_from = weight, names_prefix = "weight_d", values_fill = list(weight_d0 = 0, weight_d1 = 0))

# Step 3: Display the summary table with a title
summary_table_wide %>%
  kable(caption = "Unique New Weights by Age Category and Censoring Status")
```

Unique New Weights by Age Category and Censoring
Status

| y\_cat | weight\_d1 | weight\_d0 |
| --- | --- | --- |
| (25,30] | 0.0257601 | NA |
| (30,35] | 0.0672305 | 1 |
| (35,40] | 0.0968763 | 1 |
| (40,45] | 0.1527804 | 1 |
| (45,50] | 0.2199185 | 1 |
| (50,55] | 0.3983705 | 1 |
| (55,60] | 0.4282313 | 1 |
| (60,65] | 0.6428273 | 1 |
| (65,70] | 0.5751970 | 1 |
| (70,75] | 0.7126560 | 1 |
| (75,80] | 1.8102866 | 1 |

# Analysis

## Unweighted Cox regression

```
fit_unweighted <- coxph(Surv(y, d==1) ~prs  + cluster(family), data =  df)
summary(fit_unweighted)
```

```
## Call:
## coxph(formula = Surv(y, d == 1) ~ prs, data = df, cluster = family)
## 
##   n= 579, number of events= 320 
## 
##         coef exp(coef) se(coef) robust se      z Pr(>|z|)
## prs -0.03755   0.96315  0.08884   0.09822 -0.382    0.702
## 
##     exp(coef) exp(-coef) lower .95 upper .95
## prs    0.9631      1.038    0.7945     1.168
## 
## Concordance= 0.522  (se = 0.021 )
## Likelihood ratio test= 0.18  on 1 df,   p=0.7
## Wald test            = 0.15  on 1 df,   p=0.7
## Score (logrank) test = 0.18  on 1 df,   p=0.7,   Robust = 0.14  p=0.7
## 
##   (Note: the likelihood ratio and score tests assume independence of
##      observations within a cluster, the Wald and robust score tests do not).
```

## Weighted cohort

```
fit_wc<- coxph(Surv(y, d==1) ~prs  + cluster(family),weights=vector_weights_antoniou, data =  df)
summary(fit_wc)
```

```
## Call:
## coxph(formula = Surv(y, d == 1) ~ prs, data = df, weights = vector_weights_antoniou, 
##     cluster = family)
## 
##   n= 568, number of events= 309 
##    (11 observations deleted due to missingness)
## 
##       coef exp(coef) se(coef) robust se     z Pr(>|z|)
## prs 0.1288    1.1374   0.1459    0.1154 1.116    0.264
## 
##     exp(coef) exp(-coef) lower .95 upper .95
## prs     1.137     0.8792    0.9072     1.426
## 
## Concordance= 0.499  (se = 0.02 )
## Likelihood ratio test= 0.78  on 1 df,   p=0.4
## Wald test            = 1.25  on 1 df,   p=0.3
## Score (logrank) test = 0.78  on 1 df,   p=0.4,   Robust = 1.28  p=0.3
## 
##   (Note: the likelihood ratio and score tests assume independence of
##      observations within a cluster, the Wald and robust score tests do not).
```

## Generalized weighted cohort

```
fit_gwc<- coxph(Surv(y, d==1) ~prs  + cluster(family),weights=vector_weights_new, data =  df)
summary(fit_gwc)
```

```
## Call:
## coxph(formula = Surv(y, d == 1) ~ prs, data = df, weights = vector_weights_new, 
##     cluster = family)
## 
##   n= 579, number of events= 320 
## 
##       coef exp(coef) se(coef) robust se     z Pr(>|z|)
## prs 0.1109    1.1173   0.1456    0.1119 0.991    0.322
## 
##     exp(coef) exp(-coef) lower .95 upper .95
## prs     1.117      0.895    0.8973     1.391
## 
## Concordance= 0.497  (se = 0.019 )
## Likelihood ratio test= 0.58  on 1 df,   p=0.4
## Wald test            = 0.98  on 1 df,   p=0.3
## Score (logrank) test = 0.58  on 1 df,   p=0.4,   Robust = 1.01  p=0.3
## 
##   (Note: the likelihood ratio and score tests assume independence of
##      observations within a cluster, the Wald and robust score tests do not).
```

## Frailty model

```
fit_frailty<- emfrail(Surv(y, d==1) ~prs  + cluster(family), data =  df)
summary(fit_frailty)
```

```
## Call: 
## emfrail(formula = Surv(y, d == 1) ~ prs + cluster(family), data = df)
## 
## Regression coefficients:
##        coef exp(coef) se(coef) adj. se       z    p
## prs -0.0869    0.9167   0.1118  0.1118 -0.7773 0.44
## Estimated distribution: gamma / left truncation: FALSE 
## 
## Fit summary:
## Commenges-Andersen test for heterogeneity: p-val  4.07e-18 
## no-frailty Log-likelihood: -1838.294 
## Log-likelihood: -1784.218 
## LRT: 1/2 * pchisq(108), p-val 1.24e-25
## 
## Frailty summary:
##                    estimate lower 95% upper 95%
## Var[Z]                0.680     0.443     1.019
## Kendall's tau         0.254     0.181     0.338
## Median concordance    0.251     0.178     0.338
## E[logZ]              -0.377    -0.589    -0.237
## Var[logZ]             0.959     0.554     1.691
## theta                 1.471     0.981     2.260
## Confidence intervals based on the likelihood function
```
