# Supplementary material for: A New Inverse Probability of Selection Weighted Cox Model to Deal With Outcome‐Dependent Sampling in Survival Analysis
Source: Biom J. 2025 Jun 11;67(3):e70056. doi: 10.1002/bimj.70056 (PMC12159397; doi:10.1002/bimj.70056)
Supplement: Supplementary file 1 — Supporting Information [file BIMJ-67-e70056-s001.zip › Code for Biometrical Journal ThirdCheck/Synthetic Data Analysis/SyntheticColorectalCancerDataAnalysis.html]

Analysis of synthetic colorectal cancer dataset


# Analysis of synthetic colorectal cancer dataset

#### 2024-10-26

# Introduction

This document presents the analysis of a synthetic data based on real
colorectal cancer data. The synthetic dataset was obtained using the
function syn from synthpop R package; using parametric methods
(method=“parametric”).

# Load dataset and required functions for analysis

```
# Set the working directory to the 'Synthetic Data Analysis' folder, load dataset, and  source required scripts for weight calculation

#setwd("Synthetic Data Analysis")
data<-read.table("SyntheticDataPMS2.txt",header=T,sep="") 
source("../Simulations/prepare_data_calculate_weights.R")
```

# Preprocessing and descriptive analysis of the dataset

```
data$idi<-1:nrow(data)# add individual indicator

#Censor events at 80 or older age due tue scarcity of events (in line to what is done in the analysis with the real data)
data$d[data$y>=76]<-0
data$y[data$y>=76]<-75
```

The synthetic dataset presents a similar level of censoring (71 %)
and family size distribution than the real colorectal cancer
dataset.

```
barplot(table(table(data$famid)),xlab="Number of family members",ylab="Frequency")
```

# Construction of weights

This analysis uses population 5-year incidence rates for PMS2
carriers, calculated using data from the Netherlands in 2011 (source Netherlands
Cancer Institute and published HRs for PMS2 carriers.

```
# Define age groups as intervals for analysis, with each interval representing a 5-year age range.
breaks = c(25,30,35,40,45,50,55,60,65,70,75)
# Set population incidence rates per age group (per 100,000 individuals) as a vector.
# These values represent cancer incidence rates for each age range defined in 'breaks'.
mu_k<-c(
1.97*20.59,
3.37*20.59,
7.12*20.59,
11.98*17.06,
30.38*17.06,
60.41*3.66,
112.48*3.66,
175.45*6.92,
291.36*6.92,
405.12)/100000
```

We will now calculate the weights using both the traditional weighted
cohort approach and the newly proposed generalized weighted cohort
method.

```
# Prepare the data for analysis by using the 'Prepare_data' function.
# This function takes the dataset 'data', population incidence rates 'mu_k', and age group breaks 'breaks'.
df<-Prepare_data(dat=data, population_incidence=mu_k, breaks=breaks)
# Calculate the newly proposed weights based on the prepared data frame 'df' using the #'calculate_weights_new' function.
df$vector_weights_new<-calculate_weights_new(df)
```

```
## [1] "No negative weights"
```

```
# Calculate weights according to the Antoniou method using the prepared data frame 'df'.
df$vector_weights_antoniou<-calculate_weights_antoniou(df)
```

```
#Display Antonious weights
# Step 1: Extract unique values of y_cat, d, and vector_weights_antoniou
summary_table <- df %>%
  distinct(y_cat, d, vector_weights_antoniou) %>%
  rename(weight = vector_weights_antoniou)

# Step 2: Reshape the summary table to separate columns for each value of d
summary_table_wide <- summary_table %>%
  pivot_wider(names_from = d, values_from = weight, names_prefix = "weight_d", values_fill = list(weight_d0 = 0, weight_d1 = 0))

# Step 3: Display the summary table with a title
summary_table_wide %>%
  kable(caption = "Unique Antoniou's Weights by Age Category and Censoring Status")
```

Unique Antoniou’s Weights by Age Category and Censoring
Status

| y\_cat | weight\_d0 | weight\_d1 |
| --- | --- | --- |
| (25,30] | 1.3030949 | 0.0907152 |
| (30,35] | 1.3413855 | 0.1806749 |
| (35,40] | 1.6528942 | 0.2538352 |
| (40,45] | 1.1832521 | 0.5876827 |
| (45,50] | 0.9259582 | 1.1357432 |
| (50,55] | 1.1788838 | 0.5527904 |
| (55,60] | 0.8148238 | 2.3579590 |
| (60,65] | 0.1927444 | 2.3838667 |
| (65,70] | -0.8339931 | 3.7509896 |
| (70,75] | 0.9216610 | 1.2506849 |

```
#Display new  weights
# Step 1: Extract unique values of y_cat, d, and vector_weights_antoniou
summary_table <- df %>%
  distinct(y_cat, d, vector_weights_new) %>%
  rename(weight = vector_weights_new)

# Step 2: Reshape the summary table to separate columns for each value of d
summary_table_wide <- summary_table %>%
  pivot_wider(names_from = d, values_from = weight, names_prefix = "weight_d", values_fill = list(weight_d0 = 0, weight_d1 = 0))

# Step 3: Display the summary table with a title
summary_table_wide %>%
  kable(caption = "Unique New Weights by Age Category and Censoring Status")
```

Unique New Weights by Age Category and Censoring
Status

| y\_cat | weight\_d0 | weight\_d1 |
| --- | --- | --- |
| (25,30] | 1 | 0.0900912 |
| (30,35] | 1 | 0.1789907 |
| (35,40] | 1 | 0.2523617 |
| (40,45] | 1 | 0.5854808 |
| (45,50] | 1 | 1.1376726 |
| (50,55] | 1 | 0.5506490 |
| (55,60] | 1 | 2.3646509 |
| (60,65] | 1 | 2.4275813 |
| (65,70] | 1 | 3.9035058 |
| (70,75] | 1 | 1.2519261 |

# Analysis

## Unweighted Cox regression

```
fit_unweighted <- coxph(Surv(y, d==1) ~rs1321311  + cluster(famid), data =  df)
summary(fit_unweighted)
```

```
## Call:
## coxph(formula = Surv(y, d == 1) ~ rs1321311, data = df, cluster = famid)
## 
##   n= 191, number of events= 56 
## 
##             coef exp(coef) se(coef) robust se     z Pr(>|z|)
## rs1321311 0.4638    1.5900   0.2677    0.2938 1.579    0.114
## 
##           exp(coef) exp(-coef) lower .95 upper .95
## rs1321311      1.59     0.6289    0.8941     2.828
## 
## Concordance= 0.535  (se = 0.042 )
## Likelihood ratio test= 2.96  on 1 df,   p=0.09
## Wald test            = 2.49  on 1 df,   p=0.1
## Score (logrank) test = 3.06  on 1 df,   p=0.08,   Robust = 2.44  p=0.1
## 
##   (Note: the likelihood ratio and score tests assume independence of
##      observations within a cluster, the Wald and robust score tests do not).
```

## Weighted cohort

```
fit_wc<- try(coxph(Surv(y, d==1) ~rs1321311  + cluster(famid),weights=vector_weights_antoniou, data =  df))
```

```
## Error in coxph.fit(X, Y, istrat, offset, init, control, weights = weights,  : 
##   Invalid weights, must be >0
```

## Generalized weighted cohort

```
fit_gwc<- coxph(Surv(y, d==1) ~rs1321311  + cluster(famid),weights=vector_weights_new, data =  df)
summary(fit_gwc)
```

```
## Call:
## coxph(formula = Surv(y, d == 1) ~ rs1321311, data = df, weights = vector_weights_new, 
##     cluster = famid)
## 
##   n= 191, number of events= 56 
## 
##             coef exp(coef) se(coef) robust se     z Pr(>|z|)
## rs1321311 0.3603    1.4338   0.2409    0.2991 1.205    0.228
## 
##           exp(coef) exp(-coef) lower .95 upper .95
## rs1321311     1.434     0.6975    0.7978     2.577
## 
## Concordance= 0.518  (se = 0.042 )
## Likelihood ratio test= 2.24  on 1 df,   p=0.1
## Wald test            = 1.45  on 1 df,   p=0.2
## Score (logrank) test = 2.26  on 1 df,   p=0.1,   Robust = 1.54  p=0.2
## 
##   (Note: the likelihood ratio and score tests assume independence of
##      observations within a cluster, the Wald and robust score tests do not).
```

## Frailty model

```
fit_frailty<- emfrail(Surv(y, d==1) ~rs1321311  + cluster(famid), data =  df)
summary(fit_frailty)
```

```
## Call: 
## emfrail(formula = Surv(y, d == 1) ~ rs1321311 + cluster(famid), 
##     data = df)
## 
## Regression coefficients:
##            coef exp(coef) se(coef) adj. se     z    p
## rs1321311 0.375     1.455    0.304   0.317 1.184 0.24
## Estimated distribution: gamma / left truncation: FALSE 
## 
## Fit summary:
## Commenges-Andersen test for heterogeneity: p-val  0.128 
## no-frailty Log-likelihood: -241.864 
## Log-likelihood: -241.183 
## LRT: 1/2 * pchisq(1.36), p-val 0.122
## 
## Frailty summary:
##                    estimate lower 95% upper 95%
## Var[Z]                0.348     0.000     1.473
## Kendall's tau         0.148     0.000     0.424
## Median concordance    0.145     0.000     0.430
## E[logZ]              -0.184    -0.894     0.000
## Var[logZ]             0.415     0.000     2.975
## theta                 2.873     0.679       Inf
## Confidence intervals based on the likelihood function
```
