# Supplementary figures and images for: A New Inverse Probability of Selection Weighted Cox Model to Deal With Outcome‐Dependent Sampling in Survival Analysis
Source: Biom J. 2025 Jun 11;67(3):e70056. doi: 10.1002/bimj.70056 (PMC12159397; doi:10.1002/bimj.70056)

Scenario    ● A1    ■ A2    ▲ A3    Censoring Level    ● No    ● Medium    ● High

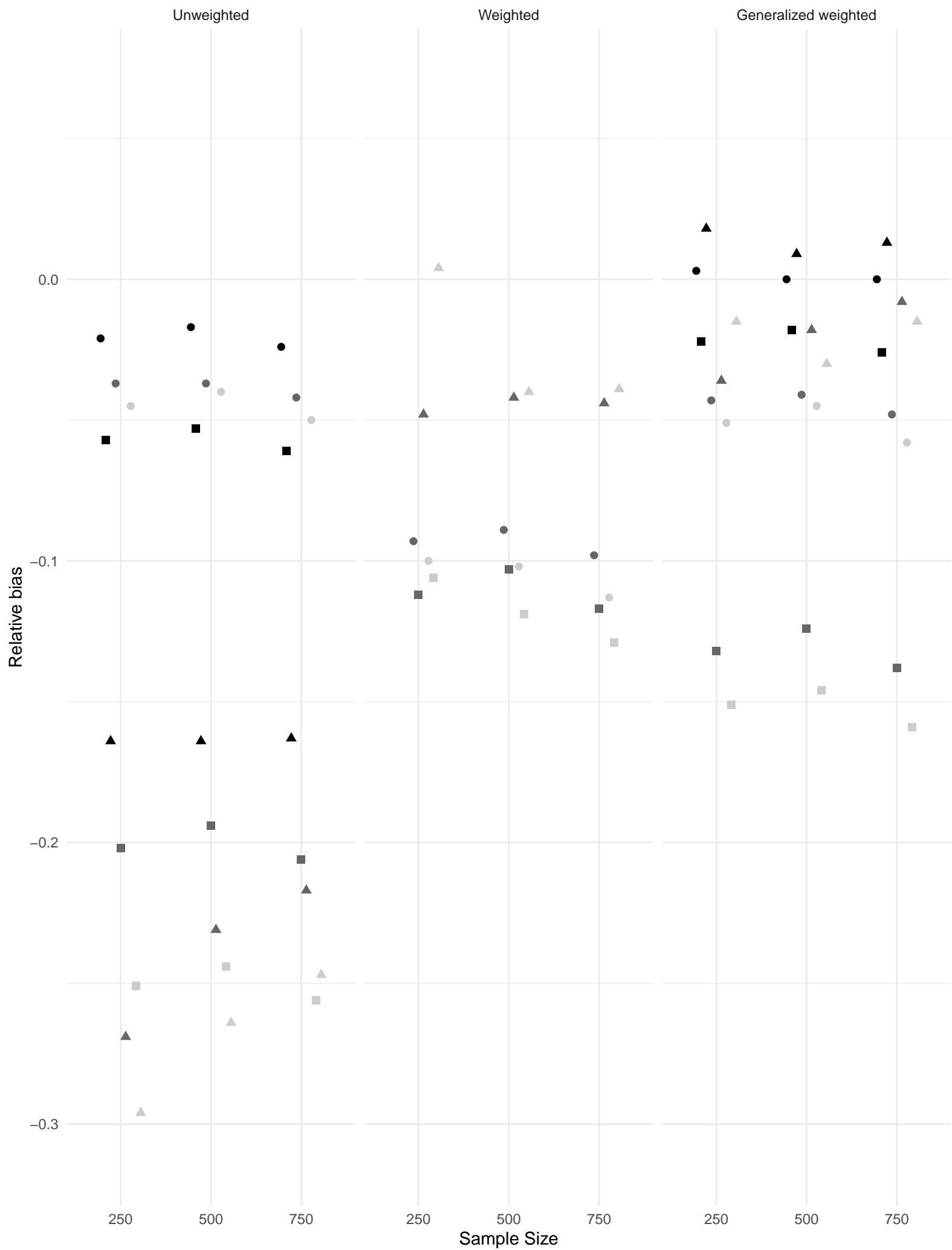

Supplement: Supplementary file 1 — Supporting Information [file BIMJ-67-e70056-s001.zip › Code for Biometrical Journal ThirdCheck/Simulations/results/figure1.pdf]

Scenario    ● A1    ■ A2    ▲ A3    Censoring Level    ● No    ● Medium    ● High

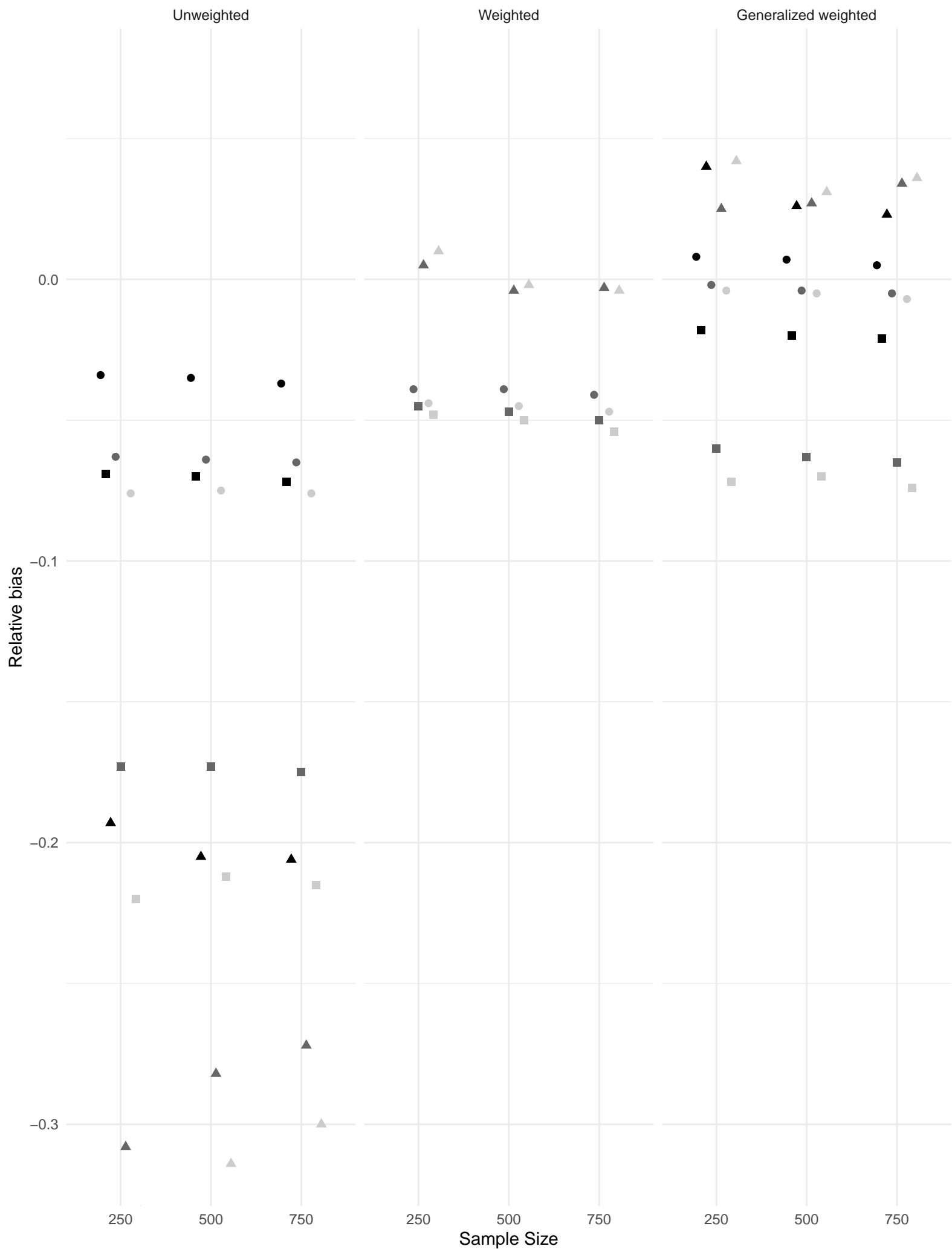

Supplement: Supplementary file 1 — Supporting Information [file BIMJ-67-e70056-s001.zip › Code for Biometrical Journal ThirdCheck/Simulations/results/figure2.pdf]
